# Exopolymeric Substances Control Microbial Community Structure and Function by Contributing to both C and Fe Nutrition in Fe-Limited Southern Ocean Provinces

**DOI:** 10.3390/microorganisms8121980

**Published:** 2020-12-12

**Authors:** Sonia Blanco-Ameijeiras, Damien J. E. Cabanes, Rachel N. Cable, Scarlett Trimborn, Stéphan Jacquet, Sonja Wiegmann, Christian Völkner, Florian Lelchat, Astrid Bracher, Melissa B. Duhaime, Christel S. Hassler

**Affiliations:** 1 Department F.-A. Forel for Environmental and Aquatic Sciences, University of Geneva—Faculty of Science, Boulevard Carl-Vogt 66, 1211 Geneva, Switzerland; sonia@blancoameijeiras.com (S.B.-A.); damien.cabanes@gmail.com (D.J.E.C.); lelchat@leoviridis.fr (F.L.); christel.hassler@epfl.ch (C.S.H.); 2 Department of Ecology and Evolutionary Biology, University of Michigan, Ann Arbor, MI 48109, USA; cabler@umich.edu (R.N.C.); duhaimem@umich.edu (M.B.D.); 3 Sections Ecological Chemistry and Physical Oceanography, Alfred Wegener Institute—Helmholtz Centre for Polar and Marine Research, Am Handelshafen 12, 27570 Bremerhaven, Germany; sonja.wiegmann@awi.de (S.W.); christian.voelkner@awi.de (C.V.); astrid.bracher@awi.de (A.B.); 4 Department Marine Botany, University of Bremen, Leobener Strasse NW2-A, 28359 Bremen, Germany; 5 INRAE, UMR CARRTEL, Université Savoie Mont-Blanc, 75bis Avenue de Corzent, 74200 Thonon-les-Bains, France ; stephan.jacquet@inrae.fr; 6 Leo Viridis, 245 rue René Descartes, 29280 Plouzané, Bretagne, France; 7 Institute of Environmental Physics, University Bremen, Otto-Hahn-Allee 1, 28359 Bremen, Germany; 8 Swiss Polar Institute, Ecole Polytechnique Fédérale de Lausanne, 1015 Lausanne, Switzerland

**Keywords:** virus, prokaryotes, phytoplankton, Southern Ocean, exopolymeric substances, iron

## Abstract

Organic ligands such as exopolymeric substances (EPS) are known to form complexes with iron (Fe) and modulate phytoplankton growth. However, the effect of organic ligands on bacterial and viral communities remains largely unknown. Here, we assessed how Fe associated with organic ligands influences phytoplankton, microbial, and viral abundances and their diversity in the Southern Ocean. While the particulate organic carbon (POC) was modulated by Fe chemistry and bioavailability in the Drake Passage, the abundance and diversity of microbes and viruses were not governed by Fe bioavailability. Only following amendments with bacterial EPS did bacterial abundances increase, while phenotypic alpha diversity of bacterial and viral communities decreased. The latter was accompanied by significantly enhanced POC, pointing toward the relief of C limitation or other drivers of the microbial loop. Based on the literature and our findings, we propose a conceptual framework by which EPS may affect phytoplankton, bacteria, and viruses. Given the importance of the Southern Ocean for Earth’s climate as well as the prevalence of viruses and their increasingly recognized impact on marine biogeochemistry and C cycling; the role of microbe–virus interactions on primary productivity in the Southern Ocean needs urgent attention.

## 1. Introduction

The activity of the lower food web (primary producers, bacteria, and archaea) is constrained by viral activity [1,2]. Through cell lysis, ocean viruses release up to 25% of the carbon fixed during primary production back to the microbial loop in dissolved form [3,4]. The role of phytoplankton and bacteria on marine biogeochemistry is well recognized and characterized including under future climate scenarios. Whereas phytoplankton is a key driver for particulate carbon (C) export [5], the microbial pump, mediated by heterotrophic bacterial transformation of dissolved organic C, is associated with a much greater C export flux [6]. Furthermore, this flux is influenced by viral infection of these central microbes [2]. Given the prevalence of viruses, their expected activity, and the great number of host-derived metabolic genes carried in viral genomes, they are thought to strongly impact marine biogeochemistry and C cycling [1,2].

The large availability of macronutrients in the Southern Ocean (SO) confers to this region a great potential for primary productivity, CO_2_ uptake, and hence Earth’s climate regulation [7]. Iron (Fe) limits the primary productivity in most of this high-nutrient low-chlorophyll *a* (Chl *a*) (HNLC) region [7,8]. Fe acts as a limiting resource for which heterotrophic bacteria and phytoplankton compete [9], while viruses as well as grazers contribute to the release of their intracellular Fe [10,11,12]. Whereas heterotrophic bacteria from the SO can be limited by either Fe or C [9,13], the controls on viral activity are less well understood. Biological activity acts as a critical linkage between the cycling of Fe and C in the ocean [6,7,14], however, few experimental field studies have attempted to explicitly link them with collected data in the field.

Together, the low concentrations of dissolved Fe, mostly as Fe associated with organic ligands (see [15] for a recent compilation of data), and the high biological requirement associated with photosynthesis [16], largely explains the Fe-limitation of primary producers in the SO. Organic ligands (L) act as key modulators for Fe speciation and solubility in seawater [14,15], affecting the bioavailability of Fe taken up by the biota to support growth and metabolism [14,17]. Concomitantly, L in the open ocean are released by phytoplankton and bacteria. These can be either Fe-binding ligands (e.g., bacterial siderophores; e.g., Velazquez et al. [18]) or by-products of metabolism, degradation, and cell lysis such as exopolymeric substances (EPS) [19,20] and refractory compounds [14]. There is evidence that EPS and carbohydrates can react with Fe and other trace metals to work as weak ligands [14,20] with implications for Fe biogeochemistry [14,21]. As EPS can also represent a significant fraction of dissolved organic C in seawater [22], EPS represents a highly relevant type of Fe-binding ligand to identify drivers for the control of microbial communities in the SO. Strong organic ligands released by heterotrophic bacteria [17] and cellular products released by viral lysis [11] can affect Fe bioavailability to phytoplankton, and one needs to include them to investigate how Fe biogeochemistry controls phytoplankton growth and biodiversity. Even free viral particles, though metabolically inert, are hypothesized to serve as Fe-binding ligands with the potential to sequester up to 70% of organic ligand-bound Fe in the surface ocean [12]. To date, limited studies have investigated how Fe associated with relevant organic compounds concomitantly control these different biological actors in the cycling of C.

In this study, we sought to address this gap by evaluating the impacts of Fe-binding ligands on the lower food web (phytoplankton, microbes, and their viruses) in the Fe-limited SO. Specifically, experiments were conducted to evaluate the impact of carbohydrates, EPS, and a siderophore using shipboard incubations of natural communities from three distinct regions of the Drake Passage (DP) and Western Antarctic Peninsula (WAP). This represents the first study south of the Polar Front to verify whether EPS enhances phytoplankton growth as reported in the Coral Sea [21] and identifies whether effects on primary producers can be related to Fe uptake rates published in a companion manuscript [23]. Moreover, we investigated how these ligands control microbial and viral abundances as well as community diversity in order to identify whether these communities were affected by changes in Fe chemistry, C sources, or changes in primary productivity.

## 2. Materials and Methods

### 2.1. Experimental Setup

All incubation bottles, tubing, reservoir carboys, and other equipment used was trace metal cleaned through methanol rinsing (99.9% Sigma-Aldrich, Buchs, Switzerland), followed by one week soaking in 0.01% Citranox (ALCONOX, White Plains, NY, USA), and one week (for polycarbonate material) or one month (for polyethylene and rest of plastics) soaking in 1.2 M HCl (VWR, Dietikon, Switzerland). After each soaking step, all materials were thoroughly rinsed with Milli-Q water (18.2 mΩ; Merck Millipore, Darmstadt, Germany) in a clean-room, and dried in a trace metal clean laminar flow hood (HEPA, class 100). Experimental setup and manipulations were conducted at sea in a trace metal clean container using trace metal clean techniques. All solutions were prepared using analytical grade chemicals (Sigma-Aldrich, Buchs, Switzerland) and Milli-Q water unless otherwise specified.

**Figure 1:**
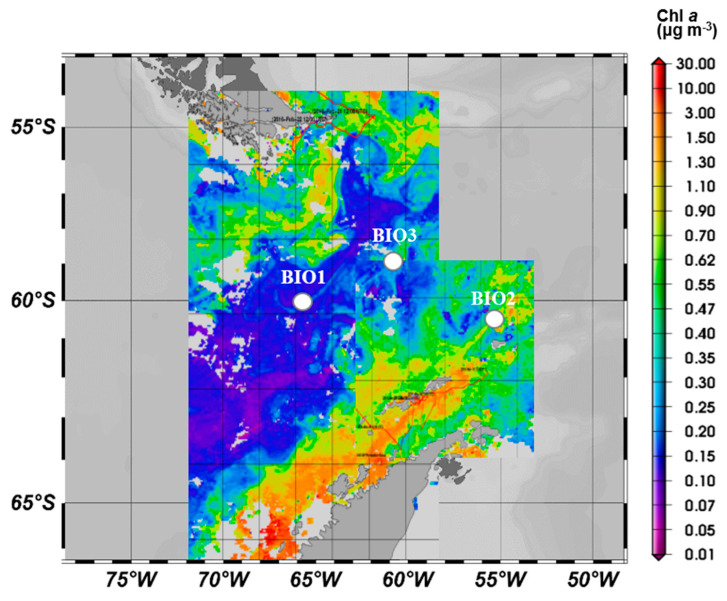
Study sites (Bio 1, 2 and 3) overlaid on the satellite-based chlorophyll *a* map. Mean chlorophyll concentrations (mg m^−3^) during March 2016 derived from the satellite MERIS Polymer product.

Experiments were performed at two sites of the DP (Bio 1 and 3) and one close to the WAP (Bio 2) during the oceanographic expedition PS97 aboard *RV Polarstern* (ANT-XXXI/3, February–March 2016) (Table 1, Figure 1). Seawater was sampled using a Teflon double diaphragm pump (Almatec, Dover Corporation, Kamp-Lintfort, Germany) coupled with a trace metal clean polyethylene tubing. Seawater was pumped directly in a laminar flow into the clean container. Prior to sampling, seawater was pumped for 1 h, allowing a thorough rinsing of the tubing. Incubation bottles were pre-rinsed three times and filled with seawater pre-filtered through a 200 µm nylon mesh to remove the mesozooplankton.

At each station, long-term (six days) incubation were setup in triplicate to test the effects of Fe enrichment either as FeCl_3_ or as FeL in the presence of six different organic ligands (L). The FeCl_3_ and FeL stock solutions used to amend seawater were prepared in advance and allowed to equilibrate for at least two days before spiking the experimental bottles. The stock solutions of organic ligands (1000-fold concentrated) included a siderophore desferrioxamine B (DFB, at 15 µM), a monosaccharide (glucuronic acid, GLU, at 22.5 mg L^−1^), a sulfated marine polysaccharide (carrageenan, CAR, at 85 mg L^−1^), and two marine acidic exopolysaccharides (L_6_, at 85 mg L^−1^; [24], and L_22_ at 85 mg L^−1^). For incubation experiments at station Bio 3, EPS isolated and concentrated (3.34-fold more concentrated than in situ, EPS1) from station Bio 1 was also tested. Concentrations of background Fe associated with organic ligands were considered in the preparation of 1000-fold concentrated FeL stock solutions to get a constant total dissolved Fe concentration (Fe_T_) of 900 nM.

The EPS L_6_ (*Cobetia marina* DSM 4741, Leibniz Institute, DSMZ-German Collection of Microorganisms and Cell Cultures GmbH) and L_22_ (*Pseudoalteromonas tunicata* DSM 14096, Leibniz Institute, DSMZ-German Collection of Microorganisms and Cell Cultures GmbH) were produced, purified, and characterized according to Lelchat et al. (2015) [24]. The native pool of EPS from Bio 1 was filtered on a 0.2 µm Acropack pressure capsule with a Supor membrane (Pall Corporation, New York, NY, USA) prior to being concentrated from 1000 L to 2 L by tangential flow filtration (Pellicon 2, Biomax 50, Cut-off 50 KDa, Merck Millipore, Darmstadt, Germany) in a trace metal room at 4 °C. All labware used for the sampling, filtration and concentration were previously cleaned and stored away from potential trace metal contamination (see above).

Incubations aimed to test the response of plankton communities to identical total Fe enrichments, but with different Fe chemistry and Fe accessibility to phytoplankton. The impact of FeL treatments on Fe chemistry and Fe uptake rates was assessed using short-term incubations (24 h) and is presented in a companion paper [23]. In this study, incubations were performed over six days at 2 °C with a 15:9 h light:dark cycle. Light was set to 35.86 ± 3.06 µmol photons m^−2^ s^−1^ by installation of a mesh with neutral density. Incubations were performed in 4 L polycarbonate bottles, enriched with FeCl_3_ or FeL under the laminar flow hood in a clean container. The bottles were gently shaken twice daily as well as prior to sampling at the end of incubation.

### 2.2. Chemical Parameters

For chemical parameters sampled at the beginning of the incubations (initial, T_0_), the seawater was 0.2 µm-filtered using an acid-cleaned Acropak 1500 capsule with a Supor membrane (Pall Corporation, New York, NY, USA) installed on the water sampling tube in the trace metal clean container. For the final sampling (after six days), filtration was performed through trace metal clean 0.2 µm polycarbonate membranes (Track-Etched, Millipore, Darmstadt, Germany) using polysulfone filtration units (Nalgene) and low vacuum pressure (<0.5 mm Hg).

*Dissolved inorganic macronutrients.* Samples for macronutrients (nitrate, nitrite, ammonium, phosphate, and dissolved silicate) were collected for each experimental bottle at the beginning and the end of incubation, 0.2 µm filtered (Sartorius Stedium, Göttingen, Germany), and stored at −20 °C in 15 mL polycarbonate vials (Falcon) until further analysis. Colorimetric determination was performed using a QuAAtro Continuous Segmented Flow Analyzer (SEAL Analytical, Norderstedt, Germany) as described in Cabanes et al. [23].

*Dissolved Fe concentrations (dFe).* dFe was determined by mass spectrometry. Samples were 0.2 µm filtered in a laminar flow hood and stored at 4 °C until analysis. Prior to analysis, all seawater samples were acidified to pH 1.7 with sub-boiled HNO_3_ (distilled 65% HNO_3_, pro analysis, Merck) and spiked with indium as the internal standard (final concentration 1 ppb). The multi-element analyses of seawater samples and process blanks were performed using a seaFAST system (Elemental Scientific Inc.) coupled to a sector field inductively coupled plasma mass spectrometer (ICP-MS, Thermo Finnigan MAT Element 2, Bremen, Germany). The commercially available seaFAST system uses a resin with ethylenediaminetriacetic acid and iminodiacetic acid functional groups to pre-concentrate metals (in this case by a factor of 40) while anions, alkali, and alkaline earth cations are washed out [25]. The ICP-MS was daily optimized to maintain oxide forming rates below 0.3% (Ba^138^/BaO^134^) and achieve a flat-top peak shape (higher precision) with a resolution of R = 2000. Quantification limit was 9.5 ng L^−1^ for ^57^Fe. A pH of 1.75 was required in order to minimize the formation of Fe hydroxides. The accuracy and precision of the method was assessed measuring the NASS-6 (National Research Council of Canada) reference standard at the beginning, in between, and also at the end of a batch run in 1:5 dilution. The recovery rate (*n* = 44) for Fe was between 100 and 102% with measured values 497 ± 46 ng L^−1^ close to certified values (495 ± 46 ng L^−1^).

### 2.3. Biological Parameters

All the biological parameters for the plankton communities, with the exception of the particulate organic matter and variable Chl *a* measurements were collected in two size fractions by sequential filtration through 10 µm and 0.6 µm using low vacuum pressure (<0.5 mm Hg). Thus, the large fraction contained cells >10 µm (L-plankton, large cells) and the small fraction contained cells between 0.6 and 10 µm (S-plankton, small cells). Data were corrected using procedural blanks and normalized to a filtered volume for all parameters.

*Particulate organic matter.* Samples for particulate organic carbon (POC) were collected at the start and the end of the incubations. To compare the contributions of large (>10 μm) and small (<10 μm) phytoplankton, POC samples were collected for the whole phytoplankton community as well as for the small phytoplankton fraction on pre-combusted GFF filters (15 h, 200 °C, ~0.6 μm, Whatman, Wisconsin, USA). To isolate the small fraction (<10μm), the samples were first passed through a 10 µm PC membrane by gravity to remove the large fraction (>10 μm), and then the filtrated was collected on the pre-combusted GFF filters for analysis. All POC filters were placed in pre-combusted glass petri dishes and immediately stored at −20 °C. Before analysis, the filters were defrosted and dried at 50 °C for 12 h, then each filter was acidified with 200 µL of 0.2 N HCl and dried again at 50 °C for 12 h. Finally, filters were packed in pre-combusted tin capsules and compressed into pellets. Samples were analyzed with an automated carbon nitrogen elemental analyzer (Euro EA 3000 CHNS-O Elemental Analyzer, HEKAtech GmbH, Wegberg, Germany). External calibration was performed using known concentrations of acetanilide (Merck, Darmstadt, Germany). Contents of POC were corrected for blank measurements and normalized to a filtered volume. Incubation time together with initial and final POC contents were used to calculate the net daily POC production rates.

*Taxonomy and Chl* a. At the start and end of the incubation experiment, aliquots of 100 mL were fixed with Lugol’s solution (1% final concentration) and stored in brown glass bottles at 4 °C in closed cardboard boxes to protect them from light. Samples for taxonomic community characterization were observed with an inverted light microscope (Axio Observer.D1; Zeiss, Oberkochen, Germany) after sedimenting for 24 h in 25 mL Utermöhl chambers (Hydro-Bios, Kiel, Germany). Qualitative analysis and species identification were done for one sample of each treatment following relevant taxonomic keys (as for [26]), and using magnifications between 100× and 640×.

To determine Chl *a* content, two size fractions (large >10 μm and small <10 μm) were collected sequentially onto 10 µm polycarbonate membranes (Millipore, Track-Etched) and 0.6 µm GFF filters. All filters were placed in 2 mL cryovials and snap frozen using liquid nitrogen and stored at −80 °C. Chl *a* concentrations were analyzed by high performance liquid chromatography (HPLC) following the method by Barlow et al. [27] adjusted to our instruments as detailed by Taylor et al. [28].

*Variable Chl* a *fluorescence measurement.* Variable Chl *a* fluorescence measurements were performed using the fast repetition rate fluorometer (FRRf, FastOcean PTX, Chelsea Technologies, Group Ltd., West Molesey, UK) coupled to a FastAct base unit (Chelsea Technologies Group Ltd., West Molesey, UK) at 2 °C. Chl *a* fluorescence measurements were performed at least 3 h after the onset of the incubation light phase and were dark-acclimated for 1 h prior to analysis. Single turnover fluorescence induction curves consisted of a saturation phase comprising 100 flashlets on a 2 μs pitch and a relaxation phase comprising 40 flashlets on a 50 μs pitch. Excitation light was produced by a block of 450, 530, and 624 nm light-emitting diodes with intensities automatically optimized for each sample. Next to the variable Chl *a* fluorescence measurement, at the start and end of the long-term incubation experiments, blank measurements were made with 0.22 µm filtered seawater. Using the software FastPro8 GUI (Chelsea Technologies Group Ltd.), all acquisitions were fitted to the biophysical model [29] and relaxation phase [30] to determine dark-adapted minimum (F_0_) and maximum (F_m_) photosystem II (PSII) fluorescence yields and PSII functional absorption cross-section (s_PSII_), the time constant for electron transport at the acceptor side of PSII (τ_Qa_), and the connectivity factor (P, dimensionless). The maximum PSII photochemical yield in the dark (F_v_/F_m_) was calculated following Equation (1):

F*_v_*/F*_m_* = (F*_m_*-F_0_)/F*_m_* (1)

*Flow cytometry (FCM).* Prokaryote- and virus-like particles (PLPs and VLPs, respectively) were counted using a FACSCalibur flow cytometer (Becton Dickinson Biosciences, Grenoble, France) equipped with an air-cooled laser providing 15 mW at 488 nm. The samples, initially fixed with glutaraldehyde for 15 min (0.5% final concentration, grade I, Merck, Darmstadt, Germany) and flash frozen with liquid N_2_ on board (−80 °C) until analysis. Once in the CYTRINON laboratory (INRA CARRTEL, Thonon, France), they were thawed at 37 °C, diluted in 0.02 µm filtered TE buffer (0.1 mM Tris-HCL and 1 mM EDTA, pH 8), and incubated with SYBR Green I (at a final 10^−4^ dilution of the commercial stock solution; Molecular Probes) for 5 min at ambient temperature, followed by 10 min at 75 °C, and then another 5 min at room temperature, prior to FCM analysis [31].

*FCM data analysis.* Raw data and statistical analyses were performed using the *PhenoFlow* (v.1.1.1; [32]), *flowCore* (v.1.48.1; [33]), and *flowFDA* (v.0.99; [34]) packages in R 3.5.3 [35]. All raw flow cytometry data and code for analysis are publicly available at https://github.com/DuhaimeLab/ps97_plp_vlp_data_analysis. Gate selections for PLPs and VLPs were determined visually from plots of log-transformed green fluorescence (excitation 488 nm; emission 530 ± 15 nm) height data (“FL1-H”) plotted against red fluorescence (>630 nm) height data (“FL3-H”). A clear break between events was identified and captured in VLP- and PLP-specific polygon gates. The coordinate (x, y) bounds for PLPs going clockwise around the polygon were (6.15, 0), (6.15, 5.9375), (7.7) and (7.0). The coordinate (x, y) bounds for VLPs going clockwise around the polygon were (5.4, 0), (5.4, 5), (6.15, 5.9375), and (6.15,0). Concentrations of VLPs and PLPs per sample were calculated using the event count inside each respective gate, the volume analyzed, and the sample dilution used for analysis. For each sample analyzed, a fingerprint of event kernel densities across 128 bins on the forward scatter height (‘FSC-H’), side scatter height (‘SSC-H’), FL1-H, and FL3-H channels was calculated using *flowFDA*. This fingerprint was analyzed for phenotypic alpha diversity metrics using *PhenoFlow*.

In order to compensate for the large variation in event counts across the PLP and VLP datasets (157–1026 and 399–2603, respectively), which were below the minimum recommended threshold of 10,000 events for phenotypic analysis [36], each sample was randomly subsampled to the minimum number of events across the respective dataset (157 for PLPs and 399 for VLPs). Alpha diversity was then calculated using 100 bootstraps. The mean diversity estimates for each sample were generated from 100 downsampling iterations. Inverse Simpson (Hill number D2) was reported, which represents the effective number of species (or here, phenotype-defined OTUs) in a sample weighted by the abundance of each phenotypic group, so that less abundant groups have a smaller effect on the metric [32,37]. Inverse Simpson was the alpha diversity metric least affected by sample size and it provided the most representative comparison at low sample sizes, thus deemed the most appropriate in this analysis.

### 2.4. Statistical Analysis

Data are given as the mean and standard deviation of the three biological replicates for all parameters except for flow cytometric data (see below) and POC production rate (*n* = 4). Significant differences at the level of 0.05 between the treatments were tested using one-way ANOVA followed by post-hoc (Holm-Sidak method) tests. Statistical analyses were performed using SigmaPlot (SysStat Software Inc., version 14.0 San Jose, CA, USA). For the photophysiology, Dunnett’s post-hoc comparison was performed using GraphPad Prism 5 for windows. Correlations amongst parameters were explored using Pearson Product Moment Correlation with a significance level of 0.05 using Sigma Plot (version 14.0).

For PLP and VLP abundance and diversity comparisons, linear mixed models (LMMs) were created from z-score scaled data to determine the fixed effects of experimental treatments relative to the final control treatments using the *lmerTest* package in R (v. 3.1-1, [38]). As flow cytometric data were collected under different conditions on multiple days (identified as “analysis groups”), the LMMs were used to evaluate how the random effects of the analysis group affected the variance within the data after the assigned fixed effects were considered. In order to estimate the influence of the analysis group on the remaining variance, the variance attributed to the analysis group was divided by the total residual variance. Five LMMs were developed for both the PLP and VLP analyses: two models with all stations combined (treatment and station assigned as fixed effects, analysis group as random effects, final control and stations Bio 1 or 2 as reference; Supplementary Tables S2, S4, S8) to account for all pairwise station comparisons and one model for each of the three individual stations (treatment assigned as fixed effect, analysis group as random effect, final control samples as reference; Supplementary Tables S3, S5, S7, and S9). Linear models of VMR data were run using the *lm* function from the R stats package [35] with treatment, station and analysis group as predictor terms.

## 3. Results

### 3.1. Nutrients and dFe Concentrations

In situ concentrations of macronutrients were high at the three investigated stations (Table 2). In general, the enrichment with organic ligands did not increase the in situ concentrations of the measured dissolved macronutrients. Only amendments with L_22_ promoted an increase of 16.5 and 14.2% of NO_3_ at Bio 1 and Bio 3 stations, respectively. After six days of incubation, the changes in macronutrient concentration represented 0.4, 2.6, 3.2, and 2.1% of the initial NO_3_, NO_2_, PO_4_, and SiO_3_, respectively, suggesting a small biomass build-up during the experiments. These high concentrations of nutrients discarded the co-limitation of macronutrients (Supplementary Table S1) during our experiments. At Bio 1, the NO_3_ concentrations increased between 1.27 and 3.64 µM (4%) during the incubation for all treatments except for L_22_, where it decreased by 1.71 µM. At Bio 2 and Bio 3, NO_3_ concentrations decreased (about 4%).

At the start of the experiment, concentrations of dFe in the control (Ctrl, no Fe addition) treatments in Bio 1, Bio 2, and Bio 3 were 0.74, 0.34, and 0.59 nM dFe, respectively. FeCl_3_ and FeL additions resulted in an increased dFe concentration of 0.91 ± 0.08 nM for Fe treatment, 0.87 ± 0.18 nM for DFB treatment, 0.81 ± 0.17 nM for L_6_ treatment, 0.97 ± 0.18 nM for GLU (glucuronic acid) treatment, 0.87 ± 0.11 nM for CAR treatment, and 1.43 ± 0.07 nM for L_22_ treatment (Table 2).

### 3.2. Plankton Community Structure

**Figure 2:**
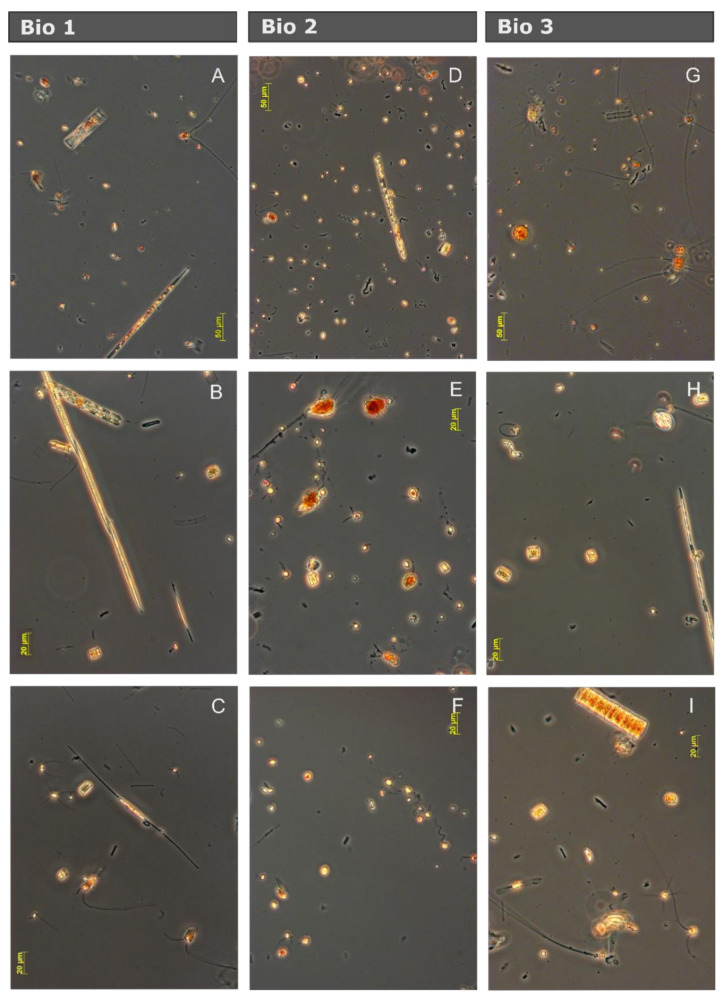
Photographs from the three natural communities of the Drake Passage and the Western Antarctic Peninsula after six days of incubation in seawater from the control treatments using inverted light microscopy. (**A**–**C**) Bio 1 community, dominated by diatoms and sporadic presence of small flagellates. (**D**–**F**) Bio 2 community, dominated by small flagellate and with abundant representation of dinoflagellates and small diatoms, and (**G**–**I**) Bio 3 community, similar to Bio 1.

Taxonomic characterization showed that the three presented plankton communities were characterized by approximately the same species. However, the dominant species differed between the three communities (Figure 2). Bio 1 and Bio 3 were both dominated by large and medium sized diatoms such as *Chaetoceros* sp., *Pseudo-nitzschia* sp., *Fragilariopsis* sp., *Rhizosolenia* sp., *Guinardia cylindrus, Corethron pennatum, Dactyliosolen tennuijunctus, Thalassiosira* sp.*, Cylindrotheca closterium,* and *Proboscia alata*. Less abundant were dinoflagellates such as *Gymnodynium* sp.*, Protoperidinium* sp*., Gyrodynium* sp., and *Heterocapsa* sp. Small flagellates belonging to the Prymnesiophyceae (*Phaeocystis antarctica*, solitary cells), the Choanoflagellidae, and some ciliates were present. The community of Bio 2 was, in contrast, dominated by small flagellates, typically prymnesiophytes, choanoflagellates, dinoflagellates, and ciliates. Additionally, some chlorophytes and cryptophytes were identified at low abundances. The same diatom species as in Bio 1 and Bio 3 were present, but in much lower concentrations. Although mesozooplankton was removed before filling the bottles, nauplius larvae, loricate (tintinids), and non-loricate ciliates were punctually detected in the samples during the microscopic characterization.

### 3.3. Plankton Biomass and Their Accumulation Rates

**Figure 3:**
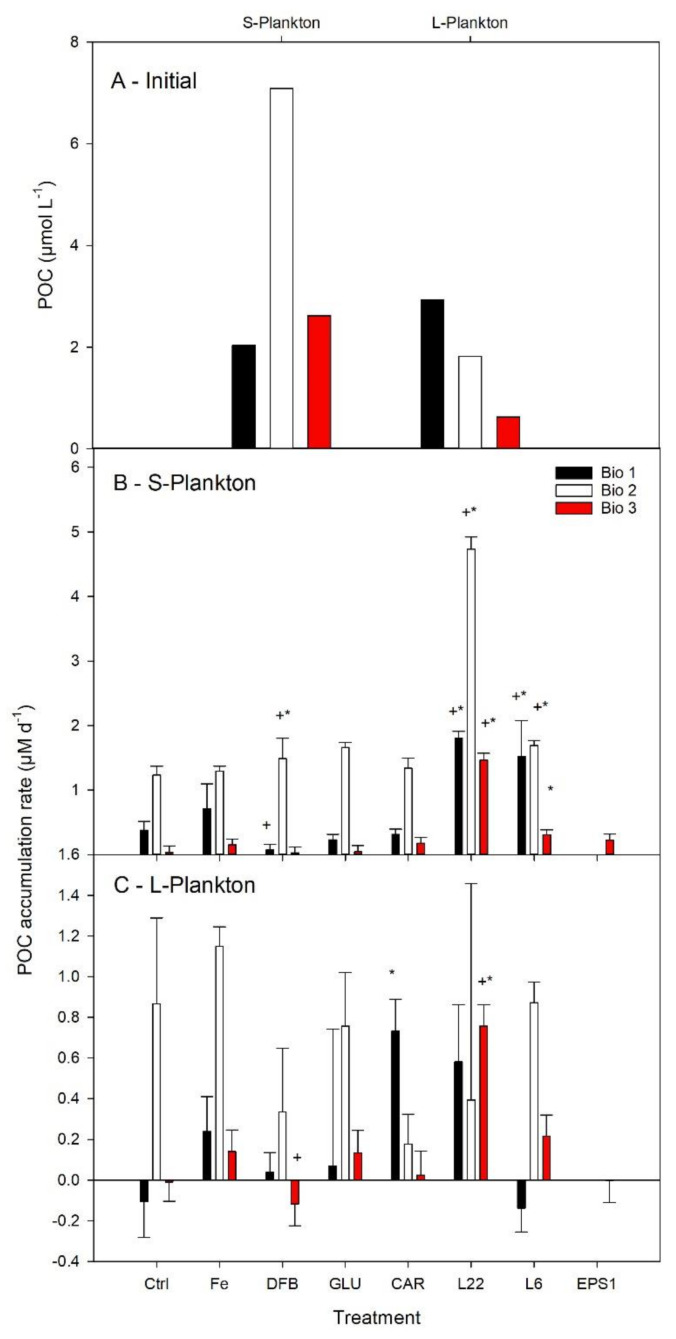
Average particulate organic carbon (POC) concentrations determined for three natural communities of the Drake Passage and the Western Antarctica Peninsula (**A**). Net daily POC accumulation rates following six days of incubation in seawater enriched with iron (Fe) bound to different organic ligands is shown for small (0.7–10 µm, S—Plankton, **B**) and large plankton (>10 µm, L—Plankton, **C**). The treatments presented are: Ctrl (control, no Fe enrichment), Fe (inorganic Fe addition, FeCl_3_), DFB (desferrioxamine B), GLU (glucuronic acid), CAR (carrageenan), L_6_ (bacterial exopolymeric substance (EPS)), L_22_ (bacterial EPS), and EPS1 (in situ EPS isolated from Bio 1). Error bars indicate standard deviation (*n* = 4). Statistical differences (level 0.05) with the Ctrl and the Fe treatments are shown by an asterisc (*) and a plus (+) symbol, respectively.

Differences in the initial POC concentrations and the relative contribution to POC by S- and L-plankton were observed in the three study sites (Figure 3A). At Bio 1, the POC content of the initial community was similar between the large and the small plankton size fraction, while at Bio 2 and Bio 3, the S-plankton was about 4-fold greater than the L-plankton size fraction.

Net daily POC accumulation rates were calculated to determine the POC-specific plankton biomass build-up of each size fraction in relation to the different experimental conditions (Figure 3B,C). In the Ctrl, the highest POC accumulation was observed for both size plankton fractions at Bio 2 and for S-plankton at Bio 1, suggesting that there was enough bioavailable Fe to sustain POC accumulation. Despite an increase in average net daily POC accumulation rates following FeCl_3_ addition, no statistical differences were measured between the Ctrl and the FeCl_3_ experimental treatments.

The POC accumulation rates in the treatments where Fe was added in association with an organic ligand were compared with the Ctrl and with the FeCl_3_ treatments (Figure 3B,C). DFB only decreased the POC accumulation rate compared to the FeCl_3_ treatment at Bio 1 for S-plankton and at Bio 3 for L-plankton. Significant enhancement of the S-plankton POC accumulation rates were measured in the presence of DFB as well as the EPS L_6_ and L_22_. The greatest rates of POC accumulation were obtained following L_22_ addition, and they were significantly greater than in all other experimental treatments. POC accumulation rates for L-plankton were less responsive to FeL additions, with only a significant increase in POC accumulation observed in the L_22_ treatment at site Bio 3 and CAR at site Bio 1.

**Figure 4:**
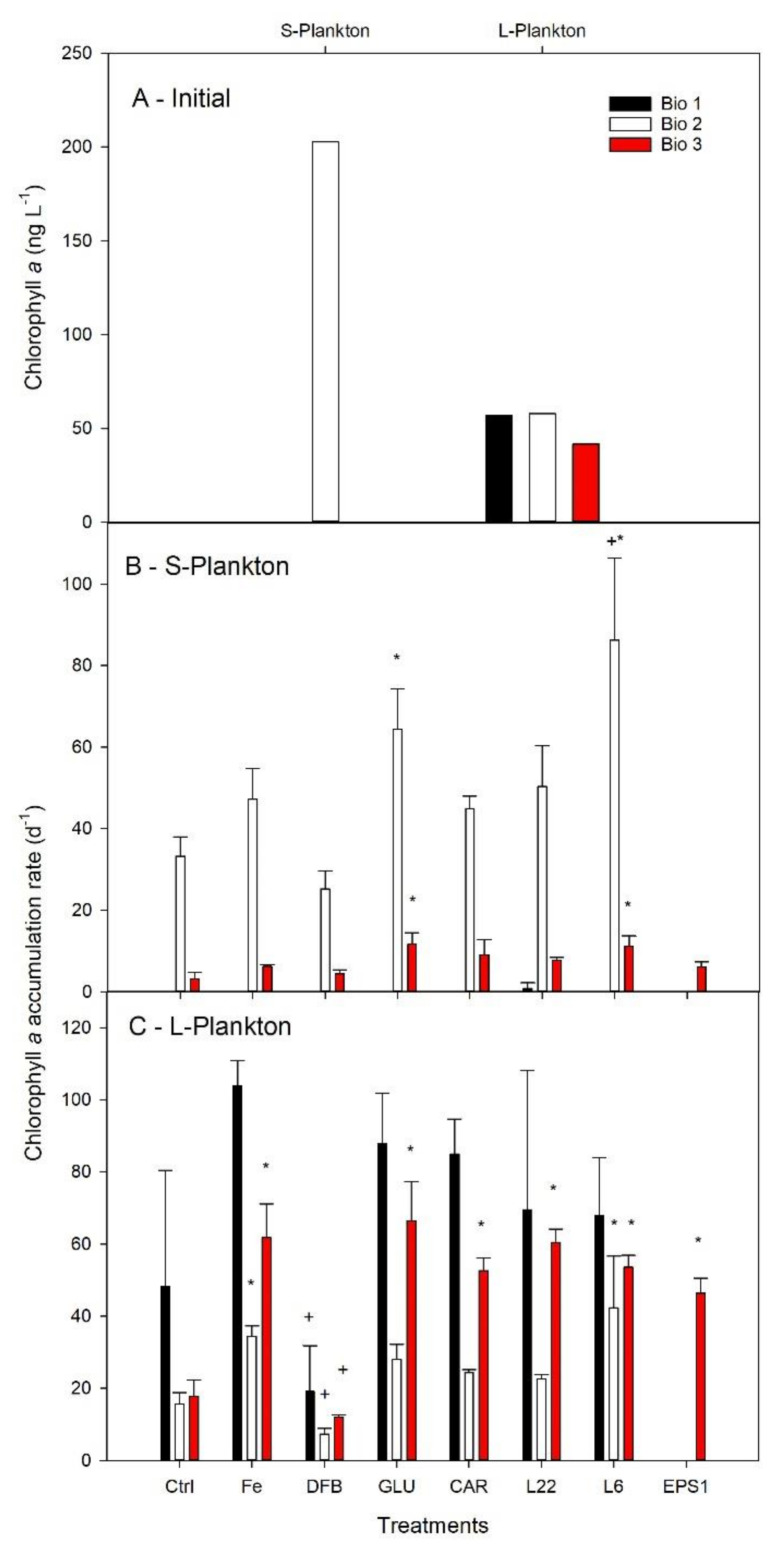
Average chlorophyll *a* concentration determined for natural communities of the Drake Passage and the West Antarctica Peninsula (**A**). Net daily chlorophyll *a* accumulation rates following six days incubation in seawater enriched with Fe (Fe) bound to different organic ligands is shown for small (0.7–10 µm, S—Plankton, **B**) and large plankton (>10 µm, L—Plankton, **C**). At Bio 3, the initial Chl *a* for S-plankton was arbitrary set to the HPLC detection limit (15 ng L^−1^) in order to estimate accumulation rates. The treatments presented are: Ctrl (control, no Fe enrichment after incubation), Fe (inorganic Fe addition, FeCl_3_), DFB (desferrioxamine B), GLU (glucuronic acid), CAR (carrageenan), L_6_ (bacterial exopolymeric substance (EPS)), L_22_ (bacterial EPS) and EPS1 (in situ EPS isolated form Bio 1). Error bars indicate standard deviation (*n* = 4). Statistical differences (level 0.05) with the Ctrl and the Fe treatments are shown by an asterisc (*) and a plus (+) symbol, respectively.

In the initial communities, the content of chlorophyll *a* (Chl *a*) was below 50 ng L^−1^ in the L-plankton for the three communities (Figure 4A). Chl *a* for the S-plankton at Bio 1 and Bio 3 was below the detection limit of the HPLC (<15 ng l^−1^), while it was about 3-fold higher in the S-plankton than for the L-plankton at Bio 2. Among Ctrl treatments, Chl *a* accumulation rates in the L-plankton were similar at all three sites, while it was highest for the S-plankton at Bio 2 (Figure 4B–C). FeCl_3_ addition further promoted Chl *a* accumulation for the L-plankton at Bio 2 and Bio 3, while it remained unchanged for the S-plankton. For L-plankton, DFB significantly decreased Chl *a* accumulation rates compared to the Fe treatment at all sites. Statistical increase compared to Ctrl was only observed at Bio 3 for GLU (both size fractions), CAR (L-plankton), L_22_ (L-plankton), L_6_ (both size fractions), EPS1 (L-plankton). Enhanced Chl *a* accumulation rates compared to Ctrl were also observed at Bio 2 for GLU (S-plankton) and L_6_ (both size fractions). It was only following L_6_ addition at Bio 2 that a greater Chl *a* accumulation rate was measured than that following FeCl_3_ enrichment.

### 3.4. Photophysiological Responses to Fe Enrichment

The maximum photochemical efficiencies (F*_v_*/F*_m_*) determined for in situ communities were 0.25 ± 0.02 and 0.23 ± 0.03 at Bio 1 and Bio 3, respectively (Table 3), while the F*_v_*/F*_m_* was higher at BIO 2 (0.47 ± 0.01). The significant increases in F*_v_*/F*_m_* and P relative to Ctrl after addition of FeCl_3_ at the three stations, suggests Fe-limiting conditions at all three sampling sites. In general, τ_Qa_ remained unaffected while ơ_PSII_ was strongly reduced in +Fe treatments relative to Ctrl treatments at Bio 1 and Bio 3. Fe-limiting conditions are further supported by the observations that at Bio 1 and Bio 3 all Fe-ligand-enrichments enhanced F*_v_*/F*_m_*, while it decreased ơ_PSII_ with respect to the Ctrl, suggesting that all ligands added were efficient to relieve Fe limitation. Only the response to Fe-DFB was less strong relative to the other ligands (Table 3). For Bio 2, this trend was much less pronounced, with an increase in F*_v_*/F*_m_* for all ligands except for DFB. Only for station Bio 2, enrichment with Fe-DFB resulted in a significant reduction of F*_v_*/F*_m_* relative to the Ctrl, indicating that this ligand increased Fe limitation to the phytoplankton community.

### 3.5. Prokaryote-Like Particles Concentration and Diversity

**Figure 5:**
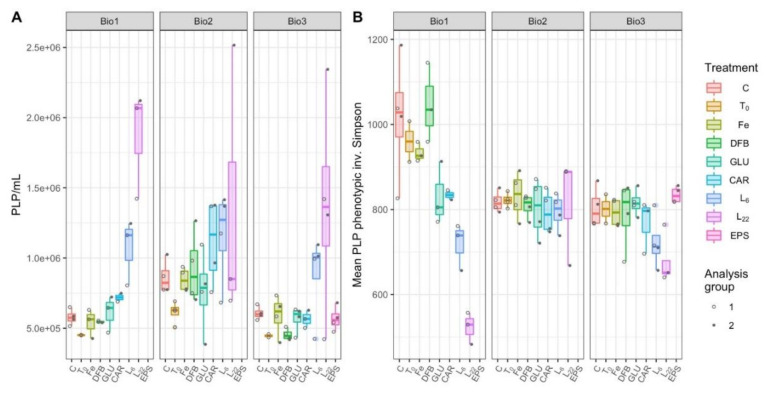
Prokaryote--like particles (PLP) concentrations (**A**) and phenotype-based inverse Simpson alpha diversity (**B**) of microbial communities during incubations with different treatments performed at each station (Bio 1–3). The treatments presented are: Ctrl (control, no Fe enrichment), Fe (inorganic Fe addition, FeCl_3_), DFB (desferrioxamine B), GLU (glucuronic acid), CAR (carrageenan), L_6_ [bacterial exopolymeric substance (EPS)], and L_22_ (bacterial EPS). Each point in panel B represents the mean of 100 bootstrapped iterations of FCM events randomly downsampled to the lowest count of gated events across the dataset.

Patterns in prokaryote-like particles (PLP) concentrations (proxy for combined bacterial and archaeal cell concentrations) at Bio 1 and Bio 3 did not differ significantly from one another (LMM, *p* = 0.1220; Figure 5A). PLP concentrations at station Bio 2 were higher than Bio 1 (*p* = 0.0652) and significantly higher than Bio 3 (*p* = 0.0004; Supplementary Table S2). Of the treatments, PLP concentrations were significantly higher than controls in the L_22_ amendments at stations Bio 1 and Bio 3 (*p* < 0.0014) and in the L_6_ treatment at Bio 1 (*p* = 0.0025; Supplementary Table S3). In this LMM, the random effects due to the analysis group accounted for only 2.7–10.9% of the variance within treatments, providing confidence that the increased PLP concentrations for L_22_ and L_6_ were attributed to treatment effects (Supplementary Table S10).

At stations Bio 1 and Bio 3, a significant reduction in microbial community diversity was observed in response to L_6_ (*p* < 0.0399) and L_22_ amendments (*p* < 0.0023), relative to control treatments (Supplementary Table S5, Figure 5B). At Bio 1, GLU (*p* = 0.0114) and CAR (*p* = 0.0252) were also significantly less diverse than controls (Supplementary Table S5). Relative to the controls, there was no impact on the microbial diversity due to any of the incubations performed at station Bio 2. The LMM indicates that differences observed at this station cannot be attributed to the treatments themselves (LMM, 106.4% residual variance attributed to analysis group; Supplementary Table S10), thus cannot be experimentally explained.

### 3.6. Virus-Like Particles Concentration and Diversity

**Figure 6:**
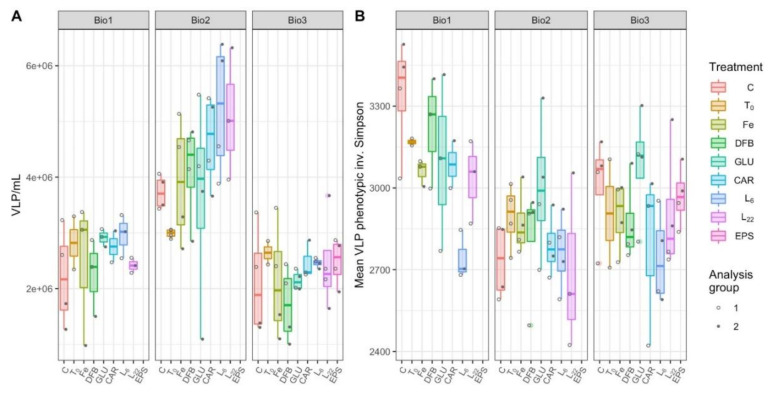
Virus-like particles (VLP) concentrations (**A**) and phenotypic biodiversity (**B**) during incubations with different treatments performed at each station (Bio 1–3). The treatments presented are: Ctrl (control, no Fe enrichment), Fe (inorganic Fe addition, FeCl_3_), DFB (desferrioxamine B), GLU (glucuronic acid), CAR (carrageenan), L_6_ (bacterial exopolymeric substance (EPS)), L_22_ (bacterial EPS). Each point in panel B represents the mean of 100 bootstrapped iterations of FCM events randomly downsampled to the lowest count of events per dataset.

As with PLPs, Bio 2 emerged as an outlier station with higher virus-like particles (VLP) concentrations than the other two stations including in the control treatments (Figure 6A; LMM, *p* < 0.0001 for Bio 1 and 3; Supplementary Table S6). There was no treatment at any station in which the VLP concentration significantly differed from the control. Incubations at Bio 2 trended toward a greater number of viruses in response to the L_6_ amendment (*p* = 0.0568; Supplementary Table S7). While this trend was statistically insignificant, none of the total variance at station Bio 2 can be attributed to random effects (Supplementary Table S10). In addition, when all stations were combined to investigate general trends, VLP concentrations in the L_6_ treatments were significantly higher than the control (*p* = 0.0130; Supplementary Table S6). In this combined station LMM, the random effect of the analysis group accounted for 15.9% of the total treatment and station variance (Supplementary Table S10).

As with the PLP phenotypic diversity, the VLP phenotypic diversity was measured using the inverse Simpson metric applied to the viral flow cytometric data (Figure 6B). In the combined station model, where 62.3% of the within treatment variance was attributed to the analysis group (Supplementary Table S10), Bio 1 had significantly higher VLP diversity than the other two stations (*p* < 0.001), and L_6_ was significantly less diverse than other treatments (*p* = 0.0005; Supplementary Table S8). This trend is supported in the individual models for Bio 1 and Bio 3, where a significant reduction in viral community diversity was observed in response to L_6_ (*p* < 0.0428; Supplementary Table S9). At Bio 1, L_22_ was also significantly less diverse than the control (*p* = 0.0401; Supplementary Table S9). At Bio 2, DFB was the only treatment significantly less diverse than the control (*p* = 0.0200; Supplementary Table S9). In the station-specific models, random effects due to the analysis group accounted for 39.4%, 82.9%, and 43.8% of the within treatment model variance, for Bio 1, 2, and 3, respectively (Supplementary Table S10).

## 4. Discussion

Our data showed contrasting responses to experimental treatments at the different sites (DP and WAP), indicating a complex relationship between Fe availability and organic ligands for natural microbial community composition [primary producers, heterotrophic prokaryotes (referred here after to prokaryotes), and viruses] across the tested SO sites. To disentangle this further, we first considered the differences in dFe concentrations and the severity of Fe limitation between WAP and DP communities. Second, we evaluated whether Fe chemistry, its bioavailability, and the potential C enrichment from the ligands added could explain our results. Based on our observations, we proposed a conceptual model, by which Fe associated to EPS can affect the microbial community in relation to Fe and C availabilities.

### 4.1. Evidence for Fe-Limitation at All Three Sampling Sites

The macronutrient concentrations measured at the investigated sites were high and in agreement with those reported in the literature for typical HNLC locations in the SO [39,40]. At Bio 1, dFe was slightly higher than expected from previous studies in this region [39,41]. This high value could be associated with deep vertical mixing due to strong winds in the Drake Passage that brings up Fe-rich waters [42] or the presence of regenerated Fe (e.g., [10]). In HNLC waters, F*_v_*/F*_m_* is generally used as an indicator of Fe limitation in many regions of the SO [39,40,41]. At the Drake Passage sampling sites Bio 1 and Bio 3, the low F*_v_*/F*_m_* values suggest severe Fe limitation (Table 3). At both sites, the in situ phytoplankton communities were dominated by large and medium sized diatoms, as previously reported in Drake Passage waters [39]. In comparison, at Bio 2, small flagellates were mainly present, with greater F*_v_*/F*_m_* indicative of intermediate Fe limitation, similar to what has been reported for coastal communities of the WAP [39,41].

Next to the photophysiological response, information on the degree of Fe limitation could also be obtained by comparing biomass buildup (POC and Chl *a*) from unamended (Ctrl) with FeCl_3_ addition (Fe). As the cellular content of Chl *a* was below the detection limits for S-plankton at Bio 1 and Bio3 and because Chl *a* could be strongly modulated by Fe availability in the absence of growth, Chl *a* accumulation rates could not be derived for all treatments and could not be unequivocally related to phytoplankton growth in our study; for these reasons Chl *a* will not be discussed hereafter. The average POC accumulation rates (Figure 3) revealed contrasting levels of Fe limitation for the different sites. At Bio 2, the average POC accumulation rates of both size fractions between Ctrl and Fe treatments remained unchanged, suggesting that Fe did not act as the main limiting nutrient. In comparison, the average POC accumulation rates increased for both size fractions after FeCl_3_ addition for the two Drake Passage sites (Bio 1 and 3) Unfortunately, high standard deviations among the triplicate incubations prevented any statistical relevance. For both stations, the L-plankton, however, shifted from an average negative to a positive POC accumulation rate following FeCl_3_ addition. POC accumulation rates did not depend solely on primary producers as prokaryotes; in particular, prokaryotes attached to biogenic particles could have contributed to the POC accumulation rates measured. Based on PLP abundances, diversity, and VLP abundances, Bio 2 strongly differed from sites at the Drake Passage; however, similar abundances and diversity for the Ctrl and Fe treatments prevent any conclusions as to whether Fe limitation acted as a key parameter for the observed differences in PLP and VLP across study sites.

During the same expedition, Fe uptake rates also support strong Fe limitation for the Drake Passage sites (Bio 1 and 3) and milder Fe limitation in the coastal WAP (Bio 2) [23]. Moreover, “mild” to strong Fe-limiting conditions at all study sites are further supported by the comparison of phytoplankton requirements for growth typically reported for Fe-limiting conditions (Kµ; Antarctic Polar Frontal zone, Indian sector, 410–450 pM, [43]; in and south of the Antarctic Polar Frontal Zone, Pacific sector, 90–110 pM, [44]) with inorganic and labile (e.g., highly reactive) Fe concentrations determined at our study sites (1–3 pM, 21–63 pM, respectively; Supplementary Table S11).

### 4.2. Response to Fe and Organic Ligands Additions by Virus, Prokaryotes, and Plankton

Whereas the response to Fe bound to various organic ligands has been largely studied for phytoplankton strains [17] and natural subantarctic phytoplankton communities [21,45], fewer studies are available for natural prokaryotic and viral communities of the SO. In this region, the relationships between bacterial production, primary production, C and Fe availability are dynamic and closely intertwined. In the SO, C, and Fe have been reported as being co-limiting for heterotrophic bacteria [9,13], with fierce competition between phytoplankton and bacteria for Fe acquisition. Moreover, such complex relationships can be affected by DOC produced by phytoplankton, which may enhance nutrient availability and bacterial growth (e.g., [46]). Biologically produced DOC can also enhance Fe acquisition and growth for primary producers [14,21]. Prokaryote communities can also impact Fe chemistry (e.g., production of strong ligands; [18]) and its bioavailability (e.g., [45]) with cascading effects on the growth of bacteria and primary producers. Given these complex relationships and feedbacks, identification of the key drivers of primary and secondary production can become challenging. Here, we compared the Fe treatments to the other FeL enrichments to better understand the role of Fe and C limitations for microbial communities in the different study regions.

### 4.3. The Response of the Plankton Community to the Different Additions

**Figure 7:**
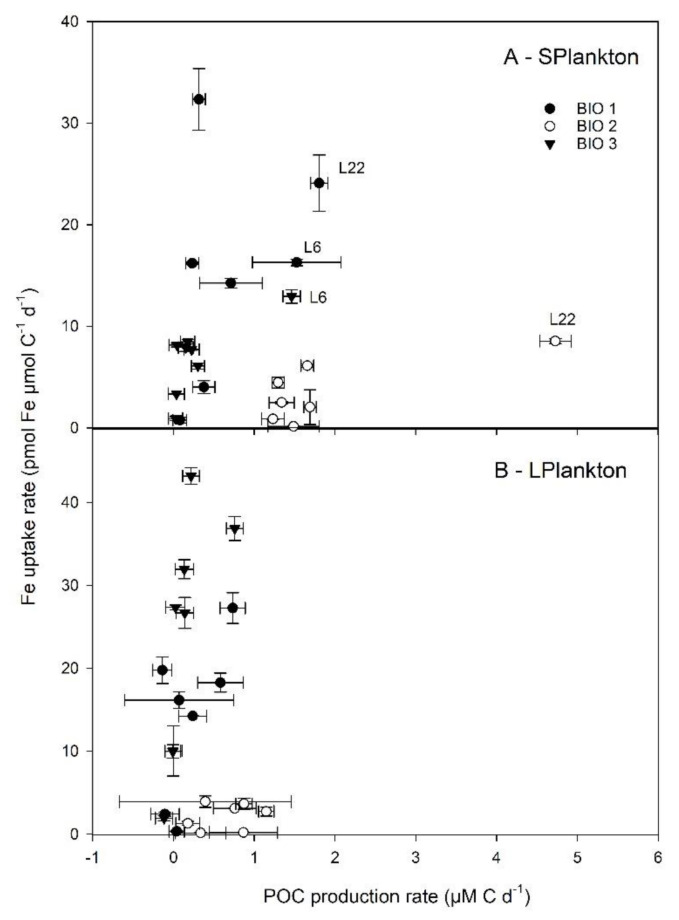
Relationship between Fe uptake rates and net daily POC production rates determined at the three stations following enrichment with Fe bound to different organic ligands. Data are shown for small (0.7–10 µm, S-Plankton, **A**) and large plankton (>10 µm, L-Plankton, **B**). Error bars indicate standard deviation (*n* = 3–4).

Based on the increased F*_v_*/F*_m_* values (Table 3), Fe added as FeCl_3_ or in association with carbohydrates and EPS was sufficient to relieve Fe-limitation, whereas Fe-DFB amendments increased the severity of Fe limitation at all sites. The distinct degree of Fe limitation and differences in phytoplankton community structures between sites Bio 1 and 3 (Drake Passage) compared to site Bio 2 (coastal WAP) could explain the differential responses of viruses, heterotrophic bacteria, and primary producers. The POC accumulation rates in the presence of excess DFB indicate a decrease in Fe availability for the Drake Passage sites, while there was an increase for the coastal WAP (Figure 3). When Fe was added in conjunction with EPS (L_6_ and L_22_), a significant enhancement was observed for S-plankton at most stations (except at Bio 3 for L_6_). For L-plankton, an enhanced POC accumulation rate was only observed for L_22_ at Bio 3. This suggests that EPS can promote natural plankton growth as previously reported in the Sub-Antarctic Zone [21]. During the same expedition as this study, Cabanes et al. [23] showed that Fe chemical speciation could explain the different Fe uptake rates determined for the same experimental treatments as used here (Supplementary Tables S11 and S12). The latter study showed that inorganic Fe concentrations (Fe’) could explain up to 70% of the Fe uptake rates measured following short-term (24 h) incubations. Here, to identify the extent by which a modulation in Fe chemistry could explain the POC accumulation rates measured, we plotted Fe uptake rates measured by Cabanes et al. [23] against the POC accumulation rates observed (this study). Overall, no significant relationships were observed between Fe uptake and POC accumulation rates (Supplementary Table S12). However, interesting phytoplankton size-specific trends were observed (Figure 7). For S-plankton, the POC accumulation rates following L_22_ and L_6_ enrichments were the greatest measured at all sites, suggesting that these organic ligands relieved other limitations (e.g., C limitation for heterotrophic bacteria, see above) and/or differently affected the microbial loop with cascading effects on biomass buildup. At site Bio 2, relatively high POC accumulation rates were achieved despite low Fe uptake rates, suggesting low Fe requirement for growth or important heterotrophic or mixotrophic contributions. At Bio 1 and Bio 3, higher net daily POC accumulation rates were observed with greater Fe uptake rates, suggesting that, despite the absence of statistical correlations, Fe was important to sustain POC production rates in the Drake Passage. Interestingly, the slope of the linear correlation between the Fe uptake rates and POC accumulation rates for Bio 1 and Bio 3 (excluding EPS experimental treatments from Figure 7), albeit not statistically relevant (*p* = 0.098 and 0.192 for L- and S- plankton, resp.), can inform on the planktonic communities’ efficiency to use Fe for POC build-up. For the latter, these slopes were similar for both plankton size classes (average of 27.2 and 20.2, for L- and S-plankton, resp.), suggesting that 20–27 pmol Fe was required to build up 1 µM C POC in the Drake Passage, corresponding to the higher range of the intracellular Fe:C ratios reported for SO phytoplankton communities and Antarctic strains (e.g., [47] for a review).

### 4.4. The Response by Prokaryotes and Virus Communities to the Different Additions

**Figure 8:**
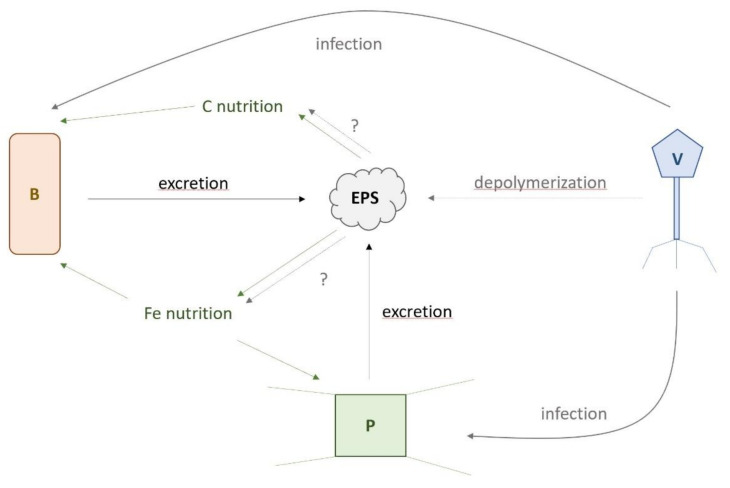
Schematic representation of potential interactions between viruses (V), prokaryotes (B), and plankton (P) in relationship to infection (blue), the role of exopolymeric substances (EPS) in carbon (C) and iron (Fe) nutrition (red), and EPS excretion as a result of cell lysis and biological activity (green). The role of viral depolymerization of EPS and their unknown impact (?) on C and Fe nutrition are shown with short dashed blue arrows.

After enrichment with Fe or in combination with organic ligands, whereby Fe chemistry was directly (FeL addition) and indirectly modulated (DOC biological production), we observed a lack of correlation between Fe chemical speciation and Fe labile concentrations (Supplementary Table S12). Our results suggest that the PLP and VLP concentrations and their diversity were not driven by Fe chemistry, but rather the amendment of C influenced the PLPs and VLPs. At the Drake Passage (Bio 1 and 3), PLP concentrations and biodiversity were significantly influenced by C amendments with the largest impact seen with EPS amendment (L_22_ and L_6,_), leading to increased PLP abundances and a concomitant decrease in their diversity (Figure 5). Hence, the addition of EPS served as a key driver for heterotrophic bacteria (Figure 8), likely relieving C limitation (e.g., [9]) in a subset of heterotrophic bacterial populations enabling them to outcompete the other bacterial populations.

However, unlike PLPs, there was no significant response of VLP abundances to EPS amendments (Figure 6A). However, there was a ligand-specific decrease in VLP diversity in response to bacterial EPS (L_6_) at Bio 1 (Figure 7B). The observed decreases in PLP and VLP diversity could be explained by different scenarios. For instance, decreased diversity can result from “Kill-the-Winner”-driven (KtW; [48]) synchrony between viral populations targeting the EPS-responsive PLP populations. However, the absence of an increase in VLP concentrations following L_6_ and L_22_ treatments (Figure 6A) suggests that if the amendments led to a bloom, the viruses targeting these populations did not lyse them during the experiment. Alternatively, the viruses of the blooming heterotrophic populations may have integrated into their host genomes upon infection, resulting in lysogeny rather than lysis. This alternate outcome of virus–host interactions in response to favorable growth conditions for host populations has been proposed as the “Piggyback-the-Winner” (PtW) model [49]. Distinguishing between these two scenarios would have required measurements of intra- and extracellular viral loads throughout the experiment. Despite this uncertainty, our study supports the undisputed position that virus–microbe–environment dynamics are inherently complex, are difficult to disentangle, and will require more targeted inquiry than community level data types such as flow cytometry are able to provide.

### 4.5. Conceptual Framework on How EPS Could Affect SO Microbial Community

Amongst our experimental treatments, the bacterial EPS was the treatment, which exhibited the greatest POC and Chl *a* accumulation rates as well as increases in PLP and VLP abundance. Together with reports that viral lysis affects Fe chemistry and its bioavailability through the release of cellular lysis products [11,12], our data demonstrated that bacterial EPS availability likely plays a role in microbe–virus infection dynamics (see Figure 8). Analysis of the correlations between viral and prokaryotic parameters (Supplementary Table S12) showed clear linkage with primary producers, specifically in relation to their biomass build-up. PLP abundances were positively correlated with POC accumulation rates of S-plankton (*p* = 0.010), while VLP abundances were positively (*p* ≤ 0.006) and diversity were negatively (*p* ≤ 0.023) correlated with POC accumulation rates of both size fractions. This suggested that PLP abundance, VLP abundance, and diversity were less influenced by Fe than the POC accumulation rate, which could be explained by the fact that phytoplankton and heterotrophic bacteria are known to excrete DOC and EPS (see references herein). Here, we propose that EPS released by excretion or cell lysis, as a result of biological activity, could constitute a link between POC build-up, PLPs, and VLPs via infection as well as C and Fe nutrition (Figure 8). Viruses use enzymes, mainly polysaccharidases, to degrade their host capsule (e.g., [50]): a prerequisite step for infection. However, these viral enzymes can also depolymerize dissolved marine bacterial EPS [51], providing a mechanism by which, in addition to cell lysis [11], viruses affect DOC chemistry with potential consequences for C and Fe bacterial and phytoplankton nutrition (dotted blue arrows, Figure 8). The impact of such virally mediated EPS depolymerization on the availability of C and Fe remains to be evaluated in the SO. The presence of bacterial EPS would have selected bacteria able to utilize this compound ([19], our study) and viruses able to infect these bacteria, but also able to degrade this EPS [51] (Figure 8). Given the important contribution of bacterial EPS to marine DOC [22] and the high abundance and turnover rate of marine viruses [1], their role in DOC transformation and alteration of Fe biogeochemistry may be considerable and require urgent assessment [52]. Our study suggested a new role for biologically-derived EPS in controlling prokaryotic and viral communities (Figure 8) and provides a framework for targeted working hypotheses for further studies.

## 5. Conclusions

Our results illustrated an important link between prokaryotes, viruses, and POC accumulation rates. However, Fe does not appear to significantly impact prokaryotic and viral abundances and diversity, though those may impact phytoplankton. This study points toward EPS as an important driver for plankton, prokaryotes, and virus community dynamics in the SO (Figure 8). However, the key drivers of community dynamics may differ amongst these groups. Our data suggest that the C source is critical for prokaryotes and viruses, in that bacterial EPS amendments appeared to select for specific prokaryotes that were able to grow using the carbon source [19]. On the other hand, it was the highly bioavailable Fe associated with the EPS, which stimulated growth of Fe-limited phytoplankton ([21,23]; this study). The manner in which prokaryotes and viruses enhance biomass build-up in the SO remains to be fully elucidated, as they likely complement the modulation of Fe bioavailability to primary producers. To better understand the interactions between plankton, prokaryotes, and viruses with consequences for the carbon cycling in the SO, a more integrative approach combining Fe and C biogeochemistry with metabolomic, metaproteomic, and metagenomic should be envisaged. Ideally, this future study should investigate natural communities as well as a previously isolated psychrophilic virus–host model system for comparison.

## Figures and Tables

**Table 1:** Details of the sampling stations during the *RV Polarstern* oceanographic expedition PS97. DP and WAP refer to the Drake Passage and Western Antarctic Peninsula, respectively.

Station	Bio 1	Bio 2	Bio 3
Region	DP	WAP	DP
Latitude	60°24.78′S	60°29.94′S	58°52.17′S
Longitude	66°21.85′W	55°29.70′W	60°51.92′W
Depth (m)	25	33	32
Date (dd.mm.yy)	03 March 2016	13 March 2016	17 March 2016

**Table 2:** Concentrations of macronutrients and Fe determined at the beginning of the incubation of each treatment: Ctrl (control), Fe (inorganic Fe addition, FeCl_3_), DFB (Fe complexed to desferrioxamine B), GLU (Fe complexed to glucuronic acid), CAR (Fe complexed to carrageenan), L_6_ (Fe complexed to bacterial exopolymeric substance, EPS), L_22_ (bacterial EPS), and EPS1 (in situ EPS isolated form Bio 1).

Station	Treatment	NO_3_ (µM)	NO_2_ (µM)	NH_4_ (µM)	PO_4_ (µM)	SiO_3_ (µM)	dFe (nM)
Bio 1	Ctrl	22.55	0.17	0.25	1.14	16.47	0.74
	Fe	21.56	0.17	0.18	1.07	14.63	1.57
	DFB	22.55	0.18	0.25	1.12	16.29	1.64
	GLU	22.29	0.17	0.13	1.12	15.98	1.59
	CAR	21.66	0.18	0.24	1.07	15.78	1.55
	L_6_	22.67	0.18	0.22	1.20	16.67	1.54
	L_22_	25.89	0.18	0.25	1.23	18.54	2.25
Bio 2	Ctrl	25.07	0.18	0.99	1.71	60.04	0.34
	Fe	25.19	0.17	0.90	1.67	56.15	1.27
	DFB	25.31	0.17	0.87	1.67	57.23	1.37
	GLU	25.25	0.18	0.86	1.70	54.61	1.42
	CAR	25.14	0.18	0.89	1.76	57.85	1.28
	L_6_	25.01	0.17	0.88	1.64	57.11	1.33
	L_22_	25.28	0.17	0.93	1.71	57.34	1.73
Bio 3	Ctrl	25.81	0.25	0.28	1.34	21.31	0.59
	Fe	25.72	0.25	0.22	1.34	21.38	1.57
	DFB	25.56	0.25	0.23	1.30	21.21	1.26
	GLU	26.77	0.26	0.25	1.54	21.75	1.48
	CAR	25.51	0.24	0.22	1.28	21.15	1.44
	L_6_	25.92	0.25	0.24	1.42	21.54	1.24
	L_22_	29.50	0.29	0.27	1.38	23.27	1.98
	EPS1	25.20	0.25	0.23	1.31	21.34	1.04

**Table 3:** The photophysiological parameters maximum photochemical efficiency (F*_v_*/F*_m_*), functional absorption cross section of photosystem II (σ_PSII_), connectivity between PSII (P), and re-oxidation time of the primary electron acceptor Q_a_ (τ_Qa_) determined for three natural communities from the Drake Passage (Bio 1 and Bio 3) and the West Antarctica Peninsula (Bio 2) measured at the day of sampling (T_0_) and after six days incubation in seawater (Ctrl = control, no Fe enrichment) and enriched with Fe (Fe = inorganic Fe addition, FeCl_3_) bound to different organic ligands (DFB = desferrioxamine B, GLU = glucuronic acid, CAR = carrageenan, L_6_ = bacterial exopolymeric substance (EPS), L_22_ = bacterial EPS, EPS1 = in situ EPS isolated form Bio 1). Significant differences (1-way ANOVA) for each parameter relative to the control are denoted by * (*p* < 0.05). ** (*p* < 0.01) and *** (*p* < 0.0001). nd denotes not determined.

Station	Treatment	F*_v_*/F*_m_* (rel. Unit)	σ_PSII_ (nm^2^ PS^−1^)	*p* (rel. Unit)	τ_Qa_ (µs)
Bio 1	T_0_	0.25 ± 0.02	8.47 ± 0.87	0.23 ± 0.05	597 ± 42
	Ctrl	0.29 ± 0.01	7.76 ± 0.06	0.21 ± 0.05	566 ± 10
	Fe	0.50 ± 0.03 ***	3.97 ± 0.18 ***	0.37 ± 0.02 ***	624 ± 19
	DFB	0.34 ± 0.04 *	5.88 ± 0.16 ***	0.21 ± 0.04	655 ± 12 *
	GLU	0.49 ± 0.01 ***	4.69 ± 0.15 ***	0.38 ± 0.02 ***	640 ± 15
	CAR	0.46 ± 0.07 ***	3.96 ± 0.35 ***	0.35 ± 0.02 ***	633 ± 27
	L_6_	0.52 ± 0.03 ***	4.52 ± 0.23 ***	0.38 ± 0.01 ***	624 ± 20
	L_22_	0.51 ± 0.01 ***	3.81 ± 0.08 ***	0.36 ± 0.01 ***	661 ± 19 *
Bio 2	T_0_	0.47 ± 0.01	4.12 ± 0.33	0.29 ± 0.05	662 ± 32
	Ctrl	0.47 ± 0.01	4.90 ± 0.82	0.26 ± 0.02	591 ± 19 ***
	Fe	0.53 ± 0.00 ***	6.27 ± 0.05 ***	0.37 ± 0.02 *	625 ± 9
	DFB	0.40 ± 0.00 ***	6.00 ± 0.09 **	0.15 ± 0.02 ***	669 ± 7
	GLU	0.49 ± 0.02	4.77 ± 0.10	0.25 ± 0.06	678 ± 18
	CAR	0.50 ± 0.01 *	4.00 ± 0.14	0.22 ± 0.05	762 ± 16 ***
	L_6_	0.53 ± 0.02 ***	4.30 ± 0.08	0.32 ± 0.01	677 ± 4
	L_22_	0.52 ± 0.00 ***	5.23 ± 0.16	0.33 ± 0.00	681 ± 14
Bio 3	T_0_	0.23 ± 0.03	4.67 ± 0.60	0.15 ± 0.02	477 ± 57
	Ctrl	0.36 ± 0.07 *	4.85 ± 0.91	nd	622 ± 72
	Fe	0.50 ± 0.01 ***	3.60 ± 0.14 *	nd	624 ± 2
	DFB	0.33 ± 0.03 *	5.18 ± 0.32	nd	609 ± 32
	GLU	0.53 ± 0.00 ***	2.67 ± 0.08 ***	nd	615 ± 5
	CAR	0.48 ± 0.02 ***	3.50 ± 0.05 **	nd	657 ± 13
	L_6_	0.51 ± 0.03 ***	2.80 ± 0.12 ***	nd	618 ± 25
	L_22_	0.52 ± 0.06 ***	2.69 ± 0.04 ***	nd	609 ± 17
	EPS1	0.50 ± 0.02 ***	2.92 ± 0.10 ***	nd	596 ± 29

## Supplementary Materials

Supplementary materials can be found at https://www.mdpi.com/2076-2607/8/12/1980/s1.
