# Supplementary material for: Exopolymeric Substances Control Microbial Community Structure and Function by Contributing to both C and Fe Nutrition in Fe-Limited Southern Ocean Provinces
Source: Microorganisms. 2020 Dec 12;8(12):1980. doi: 10.3390/microorganisms8121980 (PMC7763086; doi:10.3390/microorganisms8121980)
Supplement: Supplementary file 1 [file microorganisms-08-01980-s001.pdf]

Exopolymeric substances control microbial community structure and function by contributing to both C and Fe nutrition in Fe-limited Southern Ocean provinces

Sonia Blanco-Ameijeiras [1], Damien J. E. Cabanes [1], Rachel N. Cable [2], Scarlett Trimborn [3,4,*], Stéphan Jacquet [5], Sonja Wiegmann [3], Christian Völkner [3], Florian Lelchat [1,6], Astrid Bracher [3,7], Melissa B. Duhaime [2] and Christel S. Hassler [1,8]

[1] Department F.-A. Forel for Environmental and Aquatic Sciences, University of Geneva—Faculty of Science, Boulevard Carl-Vogt 66, 1211 Geneva, Switzerland; sonia@blancoameijeiras.com (S.B.-A.); damien.cabanes@gmail.com (D.J.E.C.); lelchat@leoviridis.fr (F.L. (F.L.); christel.hassler@epfl.ch (C.S.H.)
[2] Department of Ecology and Evolutionary Biology, University of Michigan, Ann Arbor, MI 48109, USA; cabler@umich.edu (R.N.C.); duhaimem@umich.edu (M.B.D.)
[3] Sections Ecological Chemistry and Physical Oceanography, Alfred Wegener Institute—Helmholtz Centre for Polar and Marine Research, Am Handelshafen 12, 27570 Bremerhaven, Germany; scarlett.trimborn@awi.de (S.T.); sonja.wiegmann@awi.de (S.W.); christian.voelkner@awi.de (C.V.); astrid.bracher@awi.de (A.B.)
[4] Department Marine Botany, University of Bremen, Leobener Strasse NW2-A, Bremen 28359, Germany
[5] INRAE, UMR CARRTEL, Université Savoie Mont-Blanc, 75bis Avenue de Corzent, 74200 Thonon-les-Bains, France ; stephan.jacquet@inrae.fr (S.J.)
[6] Leo Viridis, 245 rue René Descartes, Plouzané 29280, Bretagne, France
[7] Institute of Environmental Physics, University Bremen, Otto-Hahn-Allee 1, 28359 Bremen, Germany
[8] Swiss Polar Institute, Ecole Polytechnique Fédérale de Lausanne, 1015 Lausanne, Switzerland
***** Correspondence: scarlett.trimborn@awi.de; Tel.: +49-4831-1407

Supplemental information

Supplemental Table 1. Average concentrations of macronutrients and iron (Fe) determined at the end of the 6 days incubation. Values are given with standard deviation (*n*=3).

		NO_3 (µM)		NO_2 (µM)		NH_4 (µM)		PO_4 (µM)		SiO_3 (µM)		dFe (nM)	
Bio 1	Ctrl	26.19	±0.38	0.28	±0.00	0.08	±0.37	1.22	±0.02	17.59	±0.00	0.73	±0.14
	Fe	24.42	±0.49	0.26	±0.00	0.09	±0.34	1.13	±0.07	16.61	±0.00	0.83	±0.14
	DFB	23.82	±0.49	0.25	±0.00	0.00	±0.28	1.08	±0.06	16.85	±0.00	1.50	±0.08
	GLU	24.08	±0.04	0.27	±0.00	0.04	±0.15	1.20	±0.02	16.64	±0.00	0.77	±0.10
	CAR	23.66	±0.10	0.26	±0.01	0.08	±0.14	1.23	±0.01	16.52	±0.01	0.84	±0.11
	L_6	24.00	±0.79	0.26	±0.00	0.02	±0.29	1.09	±0.07	16.47	±0.00	0.75	±0.06
	L_6vd	24.21	±0.12	0.26	±0.00	0.09	±0.14	1.20	±0.04	16.36	±0.00	0.81	±0.11
	GLU	24.08	±0.04	0.27	±0.00	0.04	±0.15	1.20	±0.02	16.64	±0.00	0.77	±0.10
	CAR	23.66	±0.10	0.26	±0.01	0.08	±0.14	1.23	±0.01	16.52	±0.01	0.84	±0.11
	L_{22}	24.17	±0.06	0.27	±0.00	0.30	±0.11	1.22	±0.01	16.79	±0.00	1.01	±0.20
Bio 2	Ctrl	25.06	±0.06	0.18	±0.00	0.01	±0.01	1.45	±0.05	38.34	±0.56	0.49	±0.04
	Fe	24.61	±0.08	0.13	±0.01	0.01	±0.03	1.62	±0.02	57.40	±0.84	0.65	±0.07
	DFB	22.65	±3.02	0.11	±0.01	0.12	±0.01	1.40	±0.21	52.60	±5.94	1.22	±0.03
	GLU	24.58	±0.31	0.10	±0.01	0.17	±0.02	1.65	±0.05	57.97	±0.48	0.73	±0.13
	CAR	25.22	±1.01	0.12	±0.02	0.12	±0.02	1.69	±0.09	57.59	±1.31	0.65	±0.02
	L_6	24.80	±0.45	0.11	±0.01	0.12	±0.00	1.62	±0.04	58.69	±0.83	0.58	±0.05
	L_6vd	24.82	±0.27	0.10	±0.01	0.16	±0.03	1.63	±0.06	58.33	±0.75	0.58	±0.15
	L_{22}	24.56	±0.06	0.09	±0.01	0.11	±0.03	1.55	±0.08	56.45	±0.78	0.81	±0.09
Bio 3	Ctrl	24.36	±2.43	0.23	±0.02	0.19	±0.28	1.27	±0.07	20.41	±1.99	0.63	±0.27
	Fe	25.64	±0.25	0.24	±0.00	0.04	±0.01	1.29	±0.04	21.43	±0.27	0.60	±0.06

DFB	25.84	±0.37	0.24	±0.00	0.12	±0.01	1.32	±0.05	21.60	±0.14	1.40	±0.33
GLU	25.57	±0.96	0.24	±0.01	0.04	±0.04	1.31	±0.07	21.57	±0.88	0.61	±0.06
CAR	24.94	±0.30	0.23	±0.00	0.03	±0.02	1.32	±0.02	21.72	±0.89	0.69	±0.14
L_6	25.78	±0.98	0.24	±0.01	0.06	±0.02	1.34	±0.07	22.00	±0.74	0.63	±0.13
L_6vd	27.05	±2.93	0.25	±0.03	0.03	±0.01	1.41	±0.12	22.61	±1.86	0.60	±0.09
L_{22}	25.42	±0.33	0.23	±0.00	0.48	±0.02	1.29	±0.04	21.28	±0.25	1.09	±0.66
EPS	25.44	±0.11	0.23	±0.00	0.04	±0.03	1.28	±0.01	21.46	±0.16	0.54	±0.13

Ctrl (control), Fe (inorganic Fe addition, $FeCl_3$), DFB (iron complexed to desferrioxamine B), GLU (Fe complexed to glucuronic acid), CAR (Fe complexed to carrageenan), L_6 (Fe complexed to bacterial exopolymeric substance (EPS)), L_6vd (Fe complexed to virally degraded EPS), L_{22} (bacterial EPS) and EPS (in situ EPS isolated form Bio 1).

Supplemental Table 2. Evaluation of scaled PLP concentrations using linear mixed models fit by REML. To capture all pairwise station relationships, two models were created with all stations combined: one model with Bio 1 as the reference and a second with Bio 2 as the reference. The reference treatment was the experimental control, treatment and stations were assigned as fixed effects, and analysis group was assigned as random effect. P-values were calculated using the lmer function of the lmerTest package in R. Standard error is the standard error for the modeled intercept. Significance levels are denoted by (p < 0.1), * (p < 0.05), ** (p < 0.01), *** (p < 0.001). Model variances are reported in Supplemental Table 10.

Reference Station	Fixed Effect	Estimate	Std. Error	df	t value	Pr(>|t|)	
Bio 1	Intercept	-0.32046	0.29453	4.52453	-1.088	0.33111	
	Bio 2	0.35755	0.19109	75.12497	1.871	0.06522	.
	Bio 3	-0.30223	0.19325	75.12967	-1.564	0.12204	
Bio 2	Intercept	0.03709	0.29154	4.35112	0.127	0.904433	
	Bio 3	-0.65978	0.17910	75.00014	-3.684	0.000431	***
Bio 1 or 2	T_0	-0.26895	0.32417	75.90431	-0.83	0.40933	
	Fe	-0.01405	0.28808	75.01748	-0.049	0.96124	
	DFB	-0.06053	0.28808	75.01748	-0.21	0.83415	
	GLU	-0.05997	0.28808	75.01748	-0.208	0.83565	
	CAR	0.36047	0.30491	75.02579	1.182	0.24085	
	L_6	0.84122	0.28808	75.01748	2.92	0.00462	**
	L_{22}	2.02521	0.29578	75.06686	6.847	1.79E-09	***
	EPS 1	0.05246	0.4121	75.00347	0.127	0.89904	

Supplemental Table 3. Evaluation of scaled PLP concentrations using linear mixed models fit by REML. One model was created for each station. In all cases, the treatment reference (assigned fixed effect) was the corresponding experimental control and analysis group was assigned as random effect. P-values were calculated using the lmer function of the lmerTest package in R. Estimate is estimated intercept for the reference variable (here, the controls). Standard error is the standard error for the modeled intercept. Significance levels are denoted by ($p < 0.1$), * ($p < 0.05$), ** ($p < 0.01$), *** ($p < 0.001$). Model variances are reported in Supplemental Table 10.

Station	Treatment	Estimate	Std. Error	df	t value	Pr(>|t|)	
Bio 1	Intercept	-0.5459	0.21347	9.83593	-2.557	0.02885	*
	T_0	-0.27751	0.36273	14.96109	-0.765	0.45614	
	Fe	-0.08221	0.31797	14.20838	-0.259	0.79969	
	DFB	-0.06885	0.31797	14.20838	-0.217	0.83165	
	GLU	0.08615	0.31797	14.20838	0.271	0.79034	
	CAR	0.33005	0.36018	13.99959	0.916	0.375	
	L_6	1.16385	0.31797	14.20838	3.66	0.00252	**
	L_{22}	3.04051	0.31797	14.20838	9.562	1.43E-07	***
Bio 2	Intercept	0.11908	0.46811	8.8154	0.254	0.805	
	T_0	-0.42949	0.61301	22.88885	-0.701	0.4906	
	Fe	-0.03708	0.59983	22.00002	-0.062	0.9513	
	DFB	0.14911	0.59983	22.00002	0.249	0.806	
	GLU	-0.22919	0.59983	22.00002	-0.382	0.7061	
	CAR	0.59922	0.59983	22.00002	0.999	0.3287	
	L_6	0.70093	0.59983	22.00002	1.169	0.2551	
	L_{22}	1.20733	0.64926	22.12401	1.86	0.0763	.
Bio 3	Intercept	-0.47925	0.38204	10.50735	-1.254	0.2369	
	T_0	-0.25986	0.61482	23.65001	-0.423	0.6764	
	Fe	-0.0334	0.49568	23	-0.067	0.9469	
	DFB	-0.35744	0.49568	23	-0.721	0.4781	
	GLU	-0.09386	0.49568	23	-0.189	0.8515	
	CAR	-0.05797	0.53637	23.11358	-0.108	0.9149	
	L_6	0.64601	0.49568	23	1.303	0.2054	
	L_{22}	1.79959	0.49568	23	3.631	0.0014	**
	EPS 1	-0.09098	0.49568	23	-0.184	0.856	

Supplemental Table 4. Evaluation of scaled PLP inverse Simpson diversities using linear mixed models fit by REML. Model design and table measures are as reported in Supplemental Table 2. Model variances are reported in Supplemental Table 10. Significance levels are denoted by (p < 0.1), * (p < 0.05), ** (p < 0.01), *** (p < 0.001).

Reference Station	Fixed Effect	Estimate	Std. Error	df	t value	Pr(>|t|)	
Bio 1	Intercept	1.03134	0.26747	76	3.856	0.00024	***
	Bio 2	-0.47607	0.22278	76	-2.137	0.035818	*
	Bio 3	-0.78618	0.22527	76	-3.49	0.000808	***
Bio 2	Intercept	0.55527	0.26311	76	2.110	0.03811	*
	Bio 3	-0.31011	0.20931	76	-1.482	0.14258	
Bio 1 or 2	T_0	-0.24035	0.36905	76	-0.651	0.516845	
	Fe	-0.26763	0.33655	76	-0.795	0.428971	
	DFB	-0.07709	0.33655	76	-0.229	0.819438	
	GLU	-0.5409	0.33655	76	-1.607	0.112159	
	CAR	-0.71483	0.35616	76	-2.007	0.048301	*
	L_6	-1.14113	0.33655	76	-3.391	0.001109	**
	L_{22}	-1.82125	0.34522	76	-5.276	1.21E-06	***
	EPS 1	-0.04045	0.48157	76	-0.084	0.933288	

Supplemental Table 5. Evaluation of scaled PLP inverse Simpson diversities using linear mixed models fit by REML. Model design and table measures are as reported in Supplemental Table 3. Model variances are reported in Supplemental Table 10. Significance levels are denoted by ($p < 0.1$), * ($p < 0.05$), ** ($p < 0.01$), *** ($p < 0.001$).

Station	Treatment	Estimate	Std. Error	df	t value	Pr(>|t|)	
Bio 1	Intercept	1.844	0.3816	15	4.832	0.00022	***
	T_0	-0.5146	0.661	15	-0.779	0.448331	
	Fe	-0.7521	0.5829	15	-1.29	0.216493	
	DFB	0.2603	0.5829	15	0.447	0.661593	
	GLU	-1.6807	0.5829	15	-2.883	0.011375	*
	CAR	-1.6427	0.661	15	-2.485	0.025227	*
	L_6	-2.6751	0.5829	15	-4.589	0.000355	***
	L_{22}	-4.4292	0.5829	15	-7.598	1.61E-06	***
Bio 2	Intercept	0.05843	0.35509	1.87298	0.165	0.885	
	T_0	-0.24793	0.29364	22.19386	-0.844	0.407	
	Fe	0.13051	0.28386	22	0.46	0.65	
	DFB	-0.08722	0.28386	22	-0.307	0.762	
	GLU	-0.13537	0.28386	22	-0.477	0.638	
	CAR	-0.21785	0.28386	22	-0.767	0.451	
	L_6	-0.20613	0.28386	22	-0.726	0.475	
	L_{22}	-0.11624	0.30763	22.02045	-0.378	0.709	.
Bio 3	Intercept	-0.07062	0.23515	24	-0.3	0.76651	
	T_0	-0.02028	0.40729	24	-0.05	0.96069	
	Fe	-0.09924	0.33255	24	-0.298	0.76796	
	DFB	-0.11684	0.33255	24	-0.351	0.7284	
	GLU	0.11155	0.33255	24	0.335	0.74021	
	CAR	-0.32242	0.3592	24	-0.898	0.37832	
	L_6	-0.72252	0.33255	24	-2.173	0.03991	*
	L_{22}	-1.13584	0.33255	24	-3.416	0.00227	**
	EPS 1	0.27534	0.33255	24	0.828	0.41585	

Supplemental Table 6. Evaluation of scaled VLP concentrations using linear mixed models fit by REML. Model design and table measures are as reported in Supplemental Table 2. Model variances are reported in Supplemental Table 10. Significance levels are denoted by (p < 0.1), * (p < 0.05), ** (p < 0.01), *** (p < 0.001).

Reference Station	Fixed Effect	Estimate	Std. Error	df	t value	Pr(>|t|)	
Bio 1	Intercept	-0.627291	0.291623	4.161622	-2.151	0.0952	.
	Bio 2	1.328914	0.185529	75.115366	7.163	4.56E-10	***
	Bio 3	-0.26428	0.187624	75.119717	-1.409	0.1631	
Bio 2	Intercept	0.701623	0.288770	4.007332	2.430	0.0719	.
	Bio 3	-1.593194	0.173884	75.000128	-9.162	7.32e-14	***
Bio 1 or 2	T_0	-0.270957	0.314836	75.866898	-0.861	0.3922	
	Fe	0.107165	0.279688	75.016106	0.383	0.7027	
	DFB	-0.004714	0.279688	75.016106	-0.017	0.9866	
	GLU	0.127481	0.279688	75.016106	0.456	0.6499	
	CAR	0.512018	0.296032	75.02376	1.73	0.0878	.
	L_6	0.711635	0.279688	75.016106	2.544	0.013	*
	L_{22}	0.484968	0.28717	75.061653	1.689	0.0954	.
	EPS 1	0.45674	0.400096	75.003195	1.142	0.2573	

Supplemental Table 7. Evaluation of scaled VLP concentrations using linear mixed models fit by REML. All other measures are as in Supplemental Table 3. Model variances are reported in Supplemental Table 10. Significance levels are denoted by ($p < 0.1$), * ($p < 0.05$), ** ($p < 0.01$), *** ($p < 0.001$).

Station	Treatment	Estimate	Std. Error	df	t value	Pr(>|t|)	
Bio 1	Intercept	-0.66267	0.37143	2.38033	-1.784	0.196	
	T_0	0.2509	0.4454	14.2519	0.563	0.582	
	Fe	0.13103	0.38333	14.03968	0.342	0.738	
	DFB	-0.04644	0.38333	14.03968	-0.121	0.905	
	GLU	0.4982	0.38333	14.03968	1.3	0.215	
	CAR	0.45029	0.43284	14.00002	1.04	0.316	
	L_6	0.53698	0.38333	14.03968	1.401	0.183	
	L_{22}	0.08707	0.38333	14.03968	0.227	0.824	
Bio 2	Intercept	0.58787	0.43624	23	1.348	0.1909	
	T_0	-0.60694	0.61694	23	-0.984	0.3355	
	Fe	0.15968	0.61694	23	0.259	0.7981	
	DFB	0.32198	0.61694	23	0.522	0.6067	
	GLU	-0.08002	0.61694	23	-0.13	0.8979	
	CAR	0.76764	0.61694	23	1.244	0.2259	
	L_6	1.2374	0.61694	23	2.006	0.0568	.
	L_{22}	1.12919	0.66638	23	1.695	0.1037	
Bio 3	Intercept	-0.74244	0.315472	4.74112	-2.353	0.0681	.
	T_0	0.268035	0.444406	23.332714	0.603	0.5522	
	Fe	0.008172	0.356612	22.99997	0.023	0.9819	
	DFB	-0.328701	0.356612	22.99997	-0.922	0.3662	
	GLU	0.028362	0.356612	22.99997	0.08	0.9373	
	CAR	0.239831	0.386155	23.052655	0.621	0.5406	
	L_6	0.288277	0.356612	22.99997	0.808	0.4272	
	L_{22}	0.286956	0.356612	22.99997	0.805	0.4292	
	EPS 1	0.30761	0.356612	22.99997	0.863	0.3973	

Supplemental Table 8. Evaluation of scaled VLP inverse Simpson diversities using linear mixed models fit by REML. Model design and table measures are as reported in Supplemental Table 2. Model variances are reported in Supplemental Table 10. Significance levels are denoted by (p < 0.1), * (p < 0.05), ** (p < 0.01), *** (p < 0.001).

Reference Station	Fixed Effect	Estimate	Std. Error	df	t value	Pr(>|t|)	
Bio 1	Intercept	1.1441	0.4721	1.6887	2.424	0.158958	
	Bio 2	-1.269	0.2014	75.0335	-6.3	1.85E-08	***
	Bio 3	-0.9106	0.2037	75.0348	-4.47	2.73E-05	***
Bio 2	Intercept	-0.1249	0.4700	1.6594	-0.266	0.819694	
	Bio 3	0.3584	0.1887	75.0000	1.899	0.061437	.
Bio 1 or 2	T_0	0.2687	0.3428	75.325	0.784	0.435475	
	Fe	-0.3106	0.3036	75.0046	-1.023	0.309575	
	DFB	-0.2494	0.3036	75.0046	-0.822	0.4139	
	GLU	0.237	0.3036	75.0046	0.781	0.437432	
	CAR	-0.5604	0.3213	75.0068	-1.744	0.085248	.
	L_6	-1.0967	0.3036	75.0046	-3.612	0.000546	***
	L_{22}	-0.5401	0.3118	75.0177	-1.732	0.087327	.
	EPS 1	-0.0677	0.4343	75.0009	-0.156	0.876532	

Supplemental Table 9. Evaluation of scaled VLP inverse Simpson diversities using linear mixed models fit by REML. Model design and table measures are as reported in Supplemental Table 3. Model variances are reported in Supplemental Table 10. Significance levels are denoted by (p < 0.1), * (p < 0.05), ** (p < 0.01), *** (p < 0.001).

Station	Treatment	Estimate	Std. Error	df	t value	Pr(>|t|)	
Bio 1	Intercept	1.7846	0.4808	3.0896	3.712	0.032335	*
	T_0	-0.4703	0.6395	14.3505	-0.735	0.47397	
	Fe	-1.1299	0.5514	14.0563	-2.049	0.059568	.
	DFB	-0.4253	0.5514	14.0563	-0.771	0.453273	
	GLU	-0.9677	0.5514	14.0563	-1.755	0.101012	
	CAR	-1.112	0.6228	14	-1.786	0.095823	.
	L_6	-2.5055	0.5514	14.0563	-4.544	0.000454	***
	L_{22}	-1.2467	0.5514	14.0563	-2.261	0.040136	*
Bio 2	Intercept	1.11748	0.48313	22.2417	2.313	0.0303	*
	T_0	0.59772	0.46733	22	1.279	0.2142	
	Fe	0.35938	0.46733	22	0.769	0.4501	
	DFB	1.17263	0.46733	22	2.509	0.02	*
	GLU	0.24858	0.46733	22	0.532	0.6001	
	CAR	0.14584	0.46733	22	0.312	0.7579	
	L_6	-0.01807	0.50643	22.02577	-0.036	0.9719	
	L_{22}	-0.86455	0.5387	2.14012	-1.605	0.2417	
Bio 3	Intercept	0.3326	0.5199	3.3665	0.64	0.5633	
	T_0	-0.11	0.6698	23.236	-0.164	0.871	
	Fe	-0.4726	0.5368	23	-0.88	0.3877	
	DFB	-0.5946	0.5368	23	-1.108	0.2795	
	GLU	0.3367	0.5368	23	0.627	0.5367	
	CAR	-0.8334	0.5814	23.0366	-1.433	0.1652	
	L_6	-1.151	0.5368	23	-2.144	0.0428	*
	L_{22}	-0.4523	0.5368	23	-0.842	0.4082	
	EPS 1	-0.1668	0.5368	23	-0.311	0.7588	

Supplemental Table 10. Summary of variance analysis for all linear mixed models.

Data	Model Set	Variance due to Analysis Group	Residual Variance	Percent Residual Variance due to Analysis Group
PLP Concentration	Bio 1	0.0047	0.1730	2.7%
	Bio 2	0.0785	0.7196	10.9%
	Bio 3	0.0462	0.4914	9.4%
	All stations	0.0686	0.4754	14.4%
PLP Inverse Simpson Diversity	Bio 1	0.0000	0.5825	0.0%
	Bio 2	0.1716	0.1612	106.4%
	Bio 3	0.0000	0.2212	0.0%
	All stations	0.0000	0.6492	0.0%
VLP Concentration	Bio 1	0.1510	0.2498	60.4%
	Bio 2	0.0000	0.7612	0.0%
	Bio 3	0.0719	0.2543	28.3%
	All stations	0.0712	0.4481	15.9%
VLP Inverse Simpson Diversity	Bio 1	0.2037	0.5171	39.4%
	Bio 2	0.3620	0.4368	82.9%
	Bio 3	0.2523	0.5764	43.8%
	All stations	0.3291	0.5279	62.3%

Supplemental Table 11. Raw data for all the parameters studied. Average values with standard deviation (n= 3-4) are given.

	L		log K'Fe'L		Fe'	Felabile	Feupt L		Feupt S		POC prod L		POC prod S		PLP		PLP Inv. Simpson Div.		VLP		VLP Inv. Simpson Div.	
	nM	*Error*		*Error*	nM	nM	pM Fe µmol C^{-1} d^{-1}	*STD*	pM Fe µmol C^{-1} d^{-1}	*STD*	µM C d^{-1}	*STD*	µM C d^{-1}	*STD*	PLP mL^{-1}	*STD*	mean	*STD*	VLP mL^{-1}	*STD*	mean	*STD*
**BIO 1**																						
Cont	1.67	*0.03*	11.74	*0.02*	1.45E-03	2.80E-02	2.416	0.246	4.015	0.622	-0.106	0.176	0.377	0.137	578171.7	55935.9	1017.3	144.5	2208002.3	877814.0	3342.4	236.4
Fe	1.97	*0.02*	11.84	*0.16*	1.10E-02	2.16E-01	14.236	0.032	14.257	0.466	0.238	0.172	0.712	0.386	540035.7	103720.5	933.4	64.0	2469761.5	1301231.5	3060.3	155.8
DFB	12.50	*1.29*	12.90	*0.89*	2.50E-04		0.343	0.127	0.769	0.270	0.039	0.096	0.078	0.084	545727.3	7753.5	1046.3	103.3	2254372.2	694980.4	3222.6	231.7
GLU	1.79	*0.18*	11.72	*0.11*	1.67E-02	1.89E-01	16.153	1.005	16.203	0.097	0.069	0.673	0.232	0.083	611759.2	129976.3	829.8	79.6	2915405.4	159686.0	3097.7	313.6
CAR							27.256	1.845	32.336	3.025	0.733	0.156	0.315	0.079	718776.8	41942.4	834.0	49.4	2754528.0	399555.0	3086.2	188.6
L6	4.08	*0.16*	11.80	*0.28*	7.43E-03	1.19E-01	18.276	1.144	24.090	2.771	0.581	0.281	1.805	0.106	1070873.0	234524.2	718.8	69.8	2962476.2	390814.4	2743.4	175.6
L22	2.06	*0.01*	11.61	*0.05*	9.38E-03	1.42E-01	19.760	1.598	16.289	0.332	-0.138	0.118	1.527	0.549	1870349.2	388888.7	523.1	44.6	2416412.7	134910.7	3033.4	207.1
**BIO 2**																						
Cont	1.12	*0.10*	11.52	*0.13*	1.04E-03	2.10E-02	0.195	0.023	0.878	0.034	0.866	0.423	1.232	0.139	861464.6	118171.7	818.1	56.4	3725805.4	305854.6	2732.1	205.7
Fe	1.28	*0.10*	11.85	*0.32*	1.08E-02	2.01E-01	2.728	0.536	4.454	0.527	1.148	0.096	1.296	0.076	845669.2	83377.3	832.6	70.9	3919612.8	1112991.8	2869.8	222.7
DFB	13.30	*0.23*	13.33	*0.38*	1.20E-04		0.160	0.050	0.138	0.059	0.335	0.313	1.487	0.317	924985.5	257330.7	808.3	51.5	4116602.5	891829.9	2814.9	255.0
GLU	1.97	*0.04*	12.11	*0.14*	3.35E-03	5.90E-02	3.112	0.076	6.118	0.160	0.757	0.264	1.658	0.078	763827.2	292207.7	803.0	76.2	3628684.0	1842750.0	3002.3	293.8
CAR							1.288	0.234	2.498	0.109	0.177	0.147	1.340	0.155	1116740.6	307453.9	793.7	61.6	4657500.6	828187.9	2789.4	206.7
L6	3.21	*0.08*	12.57	*0.44*	2.48E-03	4.30E-02	3.923	0.687	8.544	0.240	0.394	1.063	4.729	0.195	1160069.5	335260.5	795.1	60.6	5227656.3	1202165.0	2765.7	206.9
L22	2.07	*0.12*	11.99	*0.29*	1.52E-03	6.80E-02	3.664	0.652	2.042	1.709	0.873	0.102	1.690	0.077	1354154.5	1009264.6	815.6	116.2	5096312.5	1185214.1	2696.7	323.5
**BIO 3**																						
Cont	1.59	*0.06*	11.29	*0.02*	5.71E-03	6.30E-02	9.988	0.795	3.345	0.059	-0.009	0.095	0.037	0.098	606568.4	46958.4	803.7	62.6	2111186.2	972594.2	3007.9	232.9
Fe	2.27	*0.09*	11.80	*0.08*	1.38E-02	2.78E-01	26.671	1.870	7.912	0.382	0.140	0.107	0.153	0.092	592337.6	143653.3	792.6	52.3	2121105.2	1038641.2	2899.0	204.8
DFB	13.30	*0.21*	12.67	*0.23*	3.30E-04		1.931	0.297	0.914	0.200	-0.117	0.108	0.027	0.087	454294.8	40578.5	790.6	82.8	1712238.3	667392.4	2870.9	205.0
GLU	1.83	*0.03*	11.50	*0.06*	1.34E-02	1.73E-01	31.929	1.136	8.155	0.175	0.133	0.112	0.044	0.099	566582.4	92304.9	816.1	56.7	2145609.3	174101.3	3085.5	243.7
CAR							27.345	0.285	8.479	0.194	0.024	0.119	0.177	0.088	565285.3	62940.9	767.7	67.2	2471404.1	345125.9	2790.5	310.1
L6	3.94	*0.09*	11.70	*0.26*	1.11E-02	1.45E-01	36.840	1.461	12.941	0.669	0.758	0.104	1.463	0.109	881776.7	308262.1	723.0	71.3	2461071.2	82144.6	2742.8	210.5
L22	1.97	*0.03*	11.79	*0.10*		1.86E-01	43.189	0.984	6.095	0.293	0.217	0.104	0.304	0.081	1373213.6	786188.6	676.9	67.1	2459468.2	861630.6	2903.7	259.4
EPS in situ							10.037	3.020	7.708	0.066	-0.003	0.107	0.225	0.093	567810.2	86368.2	834.4	53.4	2484536.4	424373.9	2969.5	204.9

Supplemental Table 12. Pearson product moment correlation amongst parameters measured. Data were obtained from Supp. Table 10 using Sigma Plot (version 14.0). The top number is the coefficient of correlation, the middle number is the p value and the bottom number is the number of observations. For significant correlations (at level of 95%), p values are shown in red.

	UptL	UptS	ProdL	ProdS	PLP	PLPDiv	VLP	VLPDiv
L	-0.341	-0.367	-0.275	-0.114	-0.23	0.217	-0.115	-0.014
(nM)	0.166	0.134	0.269	0.651	0.359	0.388	0.649	0.956
	18	18	18	18	18	18	18	18
log K'_{FeL}	-0.477	-0.396	-0.0685	0.25	-0.0832	0.272	0.296	-0.0904
	0.0453	0.104	0.787	0.317	0.743	0.275	0.232	0.721
	18	18	18	18	18	18	18	18
Fe'	0.779	0.639	-0.0457	-0.245	-0.0772	-0.283	-0.323	0.117
(nM)	0.000229	0.00579	0.862	0.344	0.768	0.272	0.206	0.654
	17	17	17	17	17	17	17	17
Fe_{labile}	0.61	0.368	-0.156	-0.426	-0.141	-0.169	-0.439	0.0799
(nM)	0.0158	0.177	0.579	0.113	0.617	0.547	0.102	0.777
	15	15	15	15	15	15	15	15
UptL		0.514	-0.0842	-0.28	0.106	-0.459	-0.472	-0.0142
(pM Fe µmol C^{-1} d^{-1})		0.0145	0.71	0.207	0.64	0.0317	0.0266	0.95
		22	22	22	22	22	22	22
UptS			0.127	0.0292	0.107	-0.274	-0.197	0.124
(pM Fe µmol C^{-1} d^{-1})			0.572	0.897	0.635	0.218	0.38	0.582
			22	22	22	22	22	22
ProdL				0.415	0.19	-0.0413	0.563	-0.483
(µmol C^{-1} d^{-1})				0.0551	0.397	0.855	0.00633	0.0229
				22	22	22	22	22
ProdS					0.539	-0.249	0.764	-0.493
(µmol C^{-1} d^{-1})					0.00964	0.263	0.0000353	0.0197
					22	22	22	22
PLP						-0.694	0.453	-0.375
(mL^{-1})						0.000343	0.0341	0.0856
						22	22	22
PLPDiv							-0.0442	0.507
							0.845	0.0161
							22	22
VLP								-0.544
(mL^{-1})								0.00879
								22
